# Lanthanum loading influences tetracycline adsorption through coupled effects of active-site generation and pore accessibility in longan peel-derived activated carbon

**DOI:** 10.1039/d6ra05083b

**Published:** 2026-07-31

**Authors:** Tra Huong Do, Thi Hue Tran, Xuan Truong Mai, Thi To Loan Nguyen, Van Nhuong Vu, Truong Xuan Vuong

**Affiliations:** a Faculty of Chemistry, Thai Nguyen University of Education No. 20 Luong Ngoc Quyen Street Thai Nguyen City 24000 Vietnam; b Faculty of Natural Sciences and Technology, TNU-University of Science Tan Thinh Ward Thai Nguyen City 24000 Vietnam xuanvt@tnus.edu.vn; c Applied Chemistry Research Group, Thai Nguyen University of Education No. 20 Luong Ngoc Quyen Street Thai Nguyen City 24000 Vietnam

## Abstract

Antibiotic residues in aquatic environments remain an important environmental concern because of their contribution to antimicrobial resistance. Although lanthanum-modified carbons have shown promising performance for tetracycline (TC) removal, the relationship between lanthanum incorporation, pore accessibility, interfacial adsorption chemistry, and adsorption performance remains poorly understood. Longan peel was converted into lanthanum-modified activated carbon through hydrothermal pretreatment, H_3_PO_4_ activation, and subsequent La_2_O_3_ incorporation. The resulting composite, prepared with a theoretical La_2_O_3_ loading of 9 wt%, exhibited a specific surface area of 264.82 m^2^ g^−1^ and achieved the highest TC removal (≈97%) among the investigated materials. Progressive La_2_O_3_ incorporation altered pore architecture and surface area, whereas adsorption performance did not increase proportionally, indicating that optimum adsorption depends on balancing structural accessibility with the development of lanthanum-containing adsorption domains. Equilibrium data were best fitted by the Sips and Toth models, with the Langmuir model estimating a maximum adsorption capacity of 91.4 mg g^−1^. XPS confirmed successful incorporation of lanthanum as surface La–O species, while lanthanum incorporation shifted the pH_p_zc from 5.5 to 7.6, consistent with modification of the interfacial charge environment. FTIR analysis, together with adsorption behavior, suggested contributions from hydrogen bonding, π–π electron donor–acceptor interactions, surface complexation, and electrostatic interactions during TC uptake. Overall, the combined structural and adsorption results suggest that adsorption performance reflects the interplay between structural accessibility and interfacial adsorption affinity. These observations support the interpretation that balancing pore architecture and surface chemistry is important for optimizing lanthanum-functionalized biomass-derived activated carbons for antibiotic removal.

## Abstract 2

Lanthanum loading balances pore accessibility and adsorption affinity for efficient TC removal.

## Graphical abstract

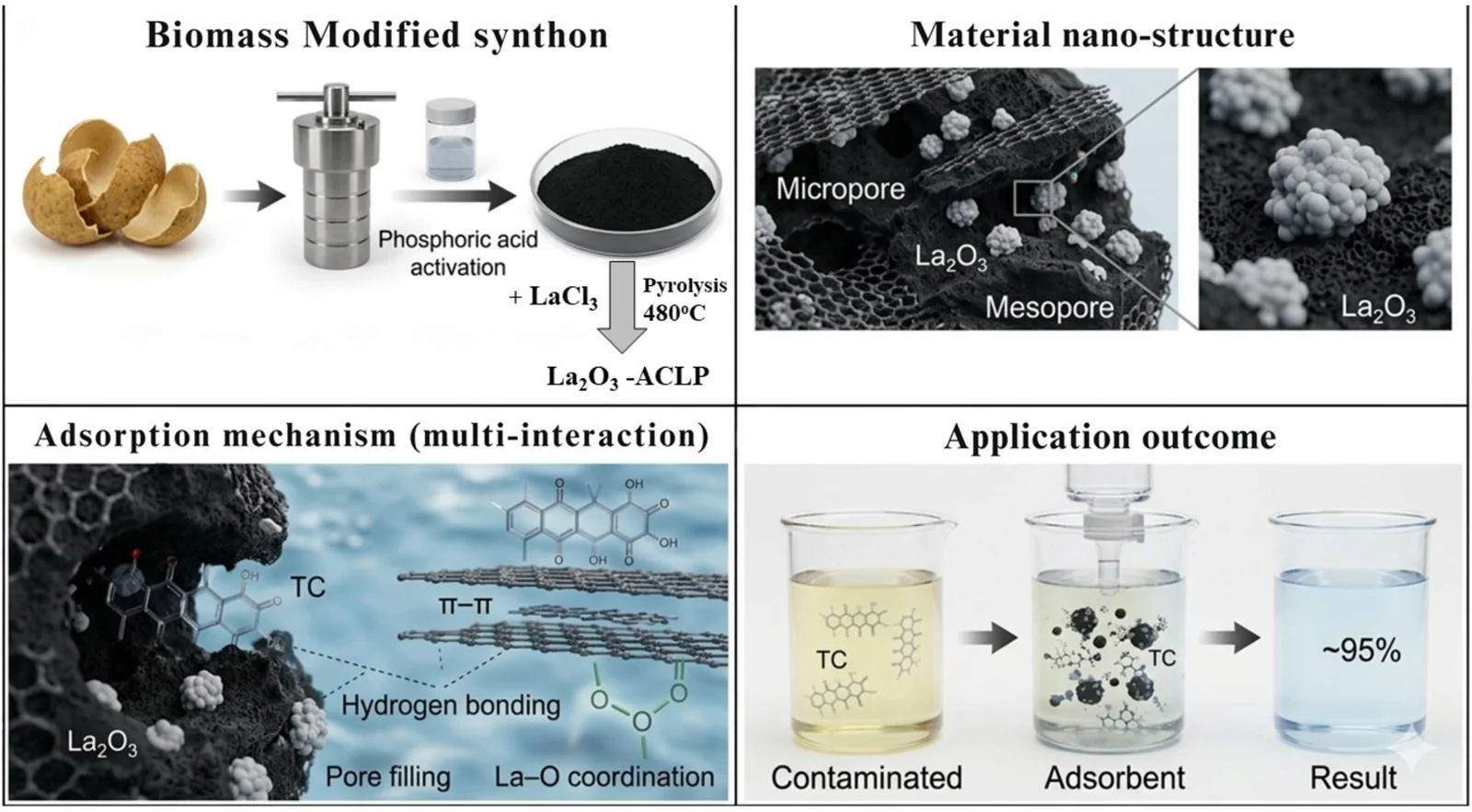

## 1. Introduction

The extensive and sustained use of antibiotics has led to their continuous release into aquatic environments, raising serious ecological and public health concerns worldwide. Among these contaminants, tetracycline (TC) remains one of the most widely used broad-spectrum antibiotics in human medicine, livestock production, and veterinary practice since its large-scale introduction in the mid-twentieth century.^1,2^ Due to incomplete metabolism and widespread consumption, a considerable portion of administered TC is excreted unchanged and subsequently enters aquatic systems through hospital effluents, municipal wastewater, livestock discharge, and agricultural runoff.^3,4^ TC has been detected in surface water, groundwater, sediments, and soils at concentrations ranging from µg L^−1^ to mg kg^−1^ levels, indicating its persistence, environmental mobility, and long-term presence in natural systems.^3,5,6^

Even at low concentrations, TC can influence microbial communities. Continuous exposure may create selective pressure, encouraging the spread of antibiotic-resistant bacteria and resistance genes.^3,4^ This phenomenon is closely tied to the broader issue of antimicrobial resistance (AMR), now regarded as a major environmental and public health challenge.^1,7^ In aquatic organisms, TC exposure has been associated with oxidative stress, growth inhibition, and metabolic disruption.^3^ These risks highlight the need for practical and environmentally compatible approaches to reduce TC contamination in water.

Various physicochemical and biological technologies have been developed for wastewater treatment, including chemical precipitation, solvent extraction, ion exchange, membrane filtration, flotation, oxidation–reduction, reverse osmosis, biosorption, and adsorption. The applicability of these technologies depends on wastewater characteristics, treatment objectives, and operational requirements. Despite these advances, the efficient removal of antibiotics remains challenging because of their persistence, structural diversity, and trace concentrations in aquatic environments. Among these technologies, advanced oxidation processes (AOPs), membrane separation, biological degradation, and adsorption have been widely investigated for antibiotic removal.^8,9^ AOPs can break down antibiotic molecules, yet their application often involves high chemical demand, elevated energy consumption, and the formation of transformation products. Membrane systems provide efficient separation but face challenges such as fouling, concentrate handling, and operational cost. Biological processes can perform well under controlled conditions, though many antibiotics resist complete biodegradation and may inhibit microbial activity. In comparison, adsorption offers a more straightforward route, combining operational simplicity with relatively low cost and rapid treatment performance when appropriate adsorbents are used.^10,11^ The success of this approach depends strongly on surface chemistry, pore structure, and the accessibility of active sites within the adsorbent.

Carbonaceous materials derived from biomass, including biochar and activated carbon, have attracted attention as sustainable adsorbents for water purification.^10,11^ Agricultural residues are widely available and inexpensive, allowing waste materials to be converted into functional products. Longan peel (*Dimocarpus longan*), generated in large quantities across Southeast Asia, is one such resource. Longan peel (*Dimocarpus longan*), generated in large quantities across Southeast Asia, is one such resource. Vietnam is one of the major longan-producing countries in Southeast Asia, with a reported production of approximately 515 100 t of fresh longan in 2014, thereby producing substantial quantities of peel as an agricultural by-product suitable as a biomass precursor for carbon materials.^12^ Its lignocellulosic structure, composed of cellulose, hemicellulose, and lignin, provides a suitable precursor for carbon-based materials. Studies have reported strong adsorption performance of longan-derived materials toward dyes and metal ions, with capacities reaching 370 mg g^−1^ for Reactive Black 5 and exceeding 500 mg g^−1^ in several dye systems.^13,14^ Additional work has demonstrated effective removal of methyl orange, methylene blue, and rhodamine B using similar materials.^2,15,16^

Despite these findings, applications targeting antibiotic removal remain limited. Most investigations involving longan-derived adsorbents focus on dyes or heavy metals, while emerging contaminants such as antibiotics receive far less attention. At the same time, lanthanum-modified carbon materials have shown encouraging performance in water treatment. Yet, systematic studies on La_2_O_3_-modified longan peel–derived activated carbon for tetracycline adsorption are still lacking. This gap suggests an opportunity to combine low-cost biomass resources with targeted surface modification to develop adsorbents tailored for pharmaceutical pollutants.

Unmodified biomass-derived carbon materials often exhibit limited surface functionality, heterogeneous active sites, and relatively weak interactions with complex organic molecules. Surface modification has therefore become a widely adopted strategy for improving the performance of adsorbent materials. Representative approaches include heteroatom doping,^17^ surface functionalization,^18^ metal oxide loading, and defect engineering,^19^ all of which can tailor the surface chemistry, electronic structure, and availability of active sites. Among these approaches, metal oxide loading has been widely investigated for adsorption applications because it can introduce additional reactive sites while retaining the porous characteristics of many carbon-based adsorbents. Lanthanum-based functionalization is of particular interest because lanthanum species interact strongly with oxygen-containing groups on carbon surfaces. Coordination with –COO^−^ and –OH groups can introduce additional adsorption sites and modify surface charge characteristics.^20^ Lanthanum-modified carbon materials have been reported to remove fluoride, phosphate, and selected metal ions through mechanisms such as electrostatic attraction, ligand exchange, surface complexation, and ion exchange.^21,22^

Recent work has extended these systems to antibiotic removal. For example, La-modified orange peel biochar exhibited a tetracycline adsorption capacity of 143.2 mg g^−1^,^23^ while La/Fe co-modified rice husk biochar reached up to 414.8 mg g^−1^.^24^ Lanthanum-based composites have also been explored for pharmaceutical removal *via* combined adsorption and catalytic pathways.^25,26^ These observations point to a strong influence of lanthanum incorporation on adsorption behavior. Yet, how such effects translate to longan peel-derived activated carbon, particularly in terms of structural modification and adsorption performance, remains unclear.

Although La-modified carbon adsorbents have been widely investigated for tetracycline removal, previous studies have focused predominantly on adsorption capacity or on demonstrating the beneficial effects of lanthanum incorporation.^23–26^ The coexistence of newly introduced lanthanum-associated adsorption sites and partial pore blockage following metal loading is therefore already well recognized.^27,28^ However, this conventional trade-off alone does not adequately explain adsorption behaviour because lanthanum incorporation simultaneously modifies pore accessibility, surface chemistry, and interfacial charge.^23,27^ Consequently, adsorption behaviour cannot be adequately interpreted from lanthanum loading or any single physicochemical characteristic alone. A systematic understanding of how these concurrent structural and surface modifications are reflected in adsorption behaviour across a controlled lanthanum-loading series remains limited.^27,28^ Addressing this question is essential for establishing an experimentally supported structure–property framework for lanthanum-functionalized biomass-derived adsorbents.

To address this knowledge gap, La_2_O_3_-modified activated carbon derived from longan peel was prepared through a combined hydrothermal-assisted phosphoric acid activation and surface-modification strategy. A controlled La_2_O_3_-loading series was established to systematically investigate the progressive evolution of pore accessibility, surface chemistry, and interfacial charge. Complementary structural characterization was interpreted together with adsorption kinetics, equilibrium, and thermodynamic analyses to examine how these concurrent physicochemical changes are reflected in adsorption behaviour. Rather than re-establishing the conventional balance between active-site generation and pore blockage, the present work provides an experimentally supported qualitative structure–property framework that relates progressive lanthanum incorporation to the collective evolution of the adsorption interface while remaining within the scope of the available experimental evidence.

The combined structural characterization and adsorption analyses provide an experimentally supported basis for interpreting how concurrent changes in pore accessibility, surface chemistry, and interfacial charge are reflected in tetracycline adsorption behaviour. Accordingly, the principal scientific contribution of this work lies in establishing this experimentally supported qualitative structure–property framework rather than solely maximizing adsorption capacity.

## 2. Materials and methods

### 2.1. Materials

All reagents used in this study were of analytical grade (≥99% purity unless otherwise specified) and employed as received without further purification to ensure experimental reproducibility.

Tetracycline (TC, ≥98%) was selected as the model antibiotic contaminant due to its widespread occurrence and environmental persistence in aqueous systems. Phosphoric acid (H_3_PO_4_, 85 wt%) was utilized as a chemical activating agent to promote pore development and surface functionalization of the carbon matrix. Lanthanum precursor was introduced in the form of lanthanum chloride heptahydrate (LaCl_3_·7H_2_O), serving as the source for *in situ* formation of La_2_O_3_ active sites. Hydrochloric acid (HCl, ∼36%), nitric acid (HNO_3_, 0.1 M), and sodium hydroxide (NaOH, 0.1 and 0.5 M) were employed for pH adjustment and precipitation control during material synthesis and adsorption experiments. All aqueous solutions were prepared using deionized water (resistivity ≥18.2 MΩ cm) to minimize interference from background ions.

### 2.2. Synthesis of La_2_O_3_-modified activated carbon derived from longan peel

Fresh longan peel (LP) was thoroughly washed with deionized (DI) water to remove adhered impurities, dried at 80 °C for 24 h, ground, and sieved to obtain particles of 1–5 mm.

#### 2.2.1 Chemical activation and hydrothermal pretreatment

The prepared LP was impregnated with 40 wt% H_3_PO_4_ at a solid-to-liquid ratio of 1 : 3 (g : mL). The mixture was stirred for 30 min and then aged at room temperature for 12 h. Subsequently, the impregnated material was transferred into a Teflon-lined stainless-steel autoclave and treated hydrothermally at 200 °C for 6 h using a solid-to-liquid ratio of 1 : 10 (g : mL).

After cooling to room temperature, the solid product was repeatedly washed with DI water until neutral pH was reached and then dried at 80 °C for 12 h.

#### 2.2.2 Preparation of activated carbon

The dried precursor was pyrolyzed in a tubular furnace under continuous nitrogen flow at 550 °C for 2 h with a heating rate of 5 °C min^−1^. After cooling under nitrogen atmosphere, the obtained activated carbon was collected and denoted as ACLP.

#### 2.2.3 Preparation of La_2_O_3_-modified composites

**Fig. 1:**
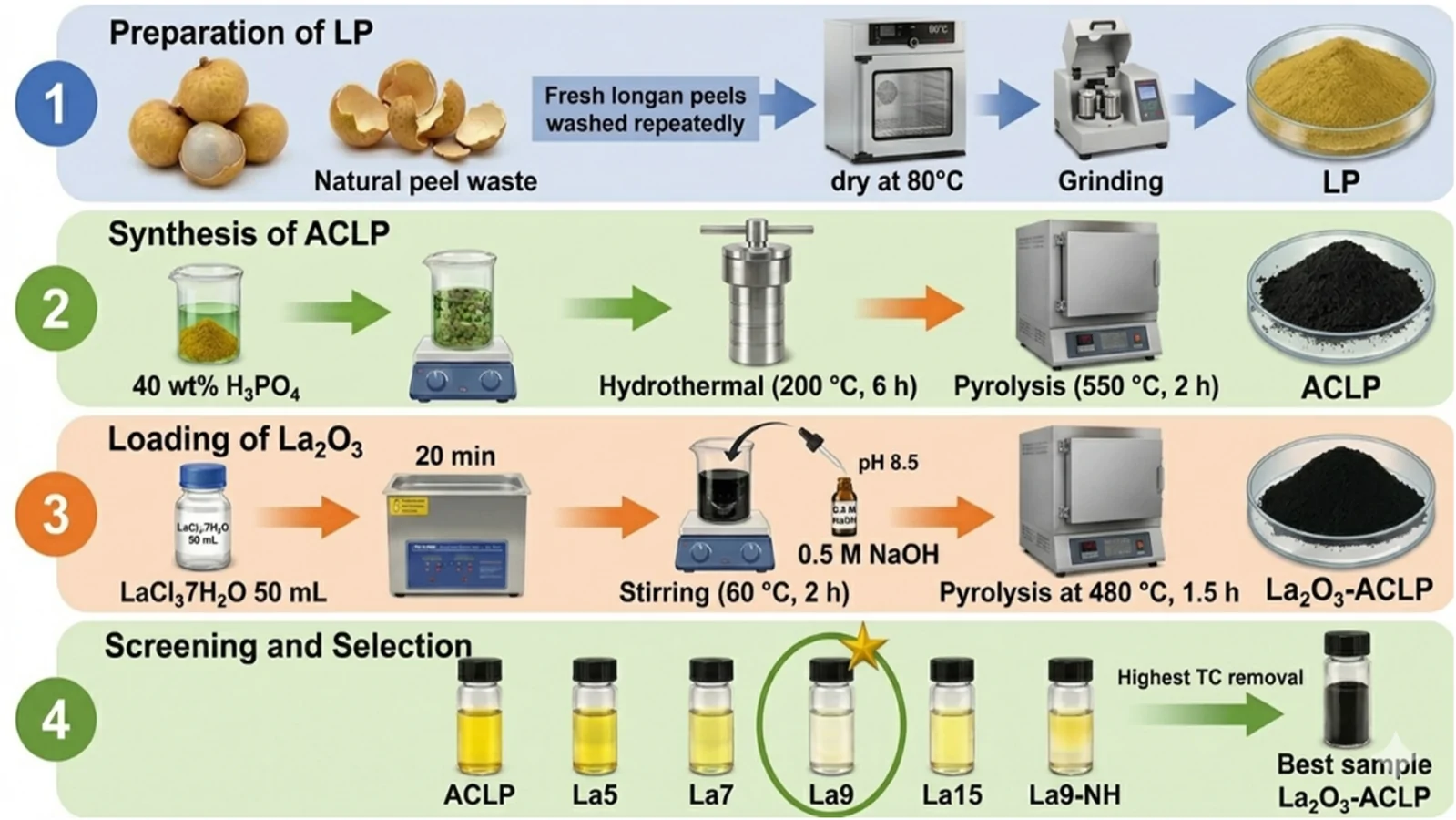
Schematic illustration of the preparation of La_2_O_3_–ACLP materials.

Lanthanum precursor solutions were prepared by dissolving 0.120–0.401 g of LaCl_3_·7H_2_O in 50 mL DI water. Then, 1.0 g of ACLP was added to each solution, followed by ultrasonication for 20 min and stirring at 60 °C for 2 h. The suspension pH was adjusted to 8.5 ± 0.2 using 0.5 M NaOH solution under continuous stirring. The mixture was further stirred for 3 h to allow deposition of lanthanum precursor species onto the carbon surface. The resulting solid was separated and washed several times with DI water until no chloride ions were detected in the filtrate using AgNO_3_ solution. The washed material was dried at 90 °C for 12 h. Calcination was then carried out under nitrogen flow at 480 °C for 1.5 h with a heating rate of 3 °C min^−1^ to obtain La_2_O_3_-modified composites. According to the theoretical La_2_O_3_ loading amounts, the prepared samples were denoted as La_5_–ACLP (or La_5_), La_7_–ACLP (La_7_), La_9_–ACLP (La_9_), and La_15_–ACLP (La_15_), corresponding to 5, 7, 9, and 15 wt% La_2_O_3_, respectively. The schematic of the preparation process is shown in Fig. 1.

#### 2.2.4 Preparation of comparative sample and sample selection

Preliminary adsorption experiments were conducted using ACLP, La_5_–ACLP (La_5_), La_7_–ACLP (La_7_), La_9_–ACLP (La_9_), La_15_–ACLP (La_15_), and La_9_–ACLP-NH (La_9_-NH) under identical conditions. Among the investigated materials, La_9_–ACLP achieved the highest TC removal efficiency (about 95%) (Fig. S1, SI) and was selected for further adsorption studies. Fig. S1 served as an initial comparative screening of La incorporation levels under identical experimental conditions. The La_9_ composition provided the most balanced adsorption response within the investigated series and was therefore selected as the representative optimum composition for subsequent detailed physicochemical characterization and adsorption analyses. These investigations were intended to elucidate the adsorption behaviour of the optimized material rather than to establish a universal mechanistic relationship across all lanthanum loadings. Accordingly, the mechanistic discussion should be regarded as a qualitative interpretation within the scope of the available experimental evidence. For clarity, this material is referred to hereafter as La_9_–ACLP or La_9_.

### 2.3. Characterization

A series of physicochemical characterization techniques was employed to investigate the morphology, composition, structural properties, surface chemistry, and thermal behavior of the synthesized materials before and after adsorption.

#### 2.3.1 Morphology and microstructure

The surface morphology of the samples was examined using scanning electron microscopy (SEM, HITACHI S-4800, Japan). Microstructural features and the distribution of inorganic particles on the carbon matrix were further observed by transmission electron microscopy (TEM, JEM-1400, JEOL, Japan).

#### 2.3.2 Elemental composition

Elemental composition and lanthanum distribution were analyzed by energy-dispersive X-ray spectroscopy (EDS) coupled with SEM (HITACHI S-4800, Japan). Elemental mapping was used to examine the spatial distribution of the main elements on the sample surface.

#### 2.3.3 Textural properties

The specific surface area, pore volume, and pore size distribution were determined from N_2_ adsorption–desorption measurements using a TriStar II 3020 analyzer (Micromeritics, USA). Prior to analysis, the samples were degassed under vacuum at 150 °C for 6 h.

#### 2.3.4 Surface functional groups

Surface functional groups of the materials before and after adsorption were identified by Fourier-transform infrared spectroscopy (FTIR, Nicolet iS10, Thermo Scientific, USA) over the wavenumber range of 400–4000 cm^−1^.

#### 2.3.5 Crystalline structure

Crystalline phases of the prepared materials were analyzed by X-ray diffraction (XRD) using a D8 Advance diffractometer (Bruker, Germany) with Cu Kα radiation (*λ* = 1.5406 Å), operated over a 2*θ* range of 5–80°.

#### 2.3.6 Carbon structural ordering

Raman spectra were recorded to evaluate the structural ordering and defect characteristics of the carbon matrix.

#### 2.3.7 Thermal behavior

Thermal stability of the materials was evaluated by thermogravimetric analysis (TGA, Labsys Evo 1600, Setaram, France). Measurements were carried out from room temperature to 800 °C at a heating rate of 10 °C min^−1^ under a controlled atmosphere.

#### 2.3.8 Surface charge characteristics (pH_pzc_ determination)

The surface charge properties of ACLP and La_9_–ACLP were evaluated by determining their point of zero charge (pH_pzc_) using the pH drift technique. Briefly, 100 mL of 0.1 M NaCl solution was adjusted to a range of initial pH values (pH_i_ = 1–10) using 0.1 M HCl or 0.1 M NaOH. Each solution was then contacted with 0.05 g of adsorbent in separate flasks.

The mixtures were allowed to equilibrate for 48 h at room temperature under intermittent shaking of the solid–liquid interface. Afterward, the final pH (pH_f_) was recorded, and the pH shift (ΔpH) was determined as the difference between final and initial values. The pH_pzc_ was identified as the pH at which ΔpH equals zero, obtained from the intersection of the ΔpH *versus* pH_i_ plot with the horizontal axis.

1 ΔpH = pH_f_ − pH

This parameter reflects the net surface charge of the adsorbent and serves as a key indicator for predicting electrostatic interactions between the material and TC species under varying pH conditions.

All measurements were conducted using independently prepared samples, and representative data are presented in the corresponding sections.

### 2.4. Adsorption experiments

Batch adsorption experiments were systematically designed to investigate the adsorption behavior of La_9_–ACLP toward tetracycline (TC), including the effects of solution pH, contact time, temperature, adsorbent dosage, and initial TC concentration. All experiments were conducted in 100 mL Erlenmeyer flasks containing 25 mL of TC solution with accurately determined initial concentrations.

#### 2.4.1 Effect of solution pH

The influence of pH on TC adsorption was first evaluated over a pH range of 2–10. In each experiment, 0.03 g of La_9_–ACLP was added to 25 mL of TC solution (25.00 mg L^−1^). The initial pH was adjusted using 0.1 M HCl or 0.1 M NaOH. The suspensions were agitated at 175 rpm for 90 min at 25 ± 1 °C. After adsorption, the mixtures were centrifuged at 4000 rpm for 10 min, followed by filtration through 0.22 µm PTFE membranes prior to analysis.

For subsequent experiments, the solution pH was fixed at 6.0 unless otherwise specified.

#### 2.4.2 Effect of contact time

To evaluate adsorption kinetics and determine equilibrium time, 0.03 g of La_9_–ACLP was added to TC solutions (20, 30, and 40 mg L^−1^). The mixtures were agitated at 175 rpm at 25 ± 1 °C over a contact time range of 30–210 min.

#### 2.4.3 Effect of temperature

The influence of temperature was assessed at 298, 313, and 323 K using 0.03 g of adsorbent and TC solution (∼30 mg L^−1^). The suspensions were shaken at 175 rpm for contact times ranging from 30 to 120 min.

#### 2.4.4 Effect of adsorbent dosage

The adsorbent dosage was varied from 0.01 to 0.07 g under constant conditions (TC concentration ∼30 mg L^−1^, pH 6.0, contact time 90 min, 25 ± 1 °C).

#### 2.4.5 Effect of initial TC concentration

Adsorption isotherm experiments were performed by varying the initial TC concentration from 15.12 to 233.06 mg L^−1^ at a fixed adsorbent dosage (0.03 g), pH 6.0, contact time 90 min, and temperature of 25 ± 1 °C.

After adsorption, all suspensions were centrifuged at 4000 rpm for 10 min and filtered through 0.22 µm PTFE membranes. The residual TC concentration was quantified using HPLC at a detection wavelength of 358 nm.

All experiments were conducted in triplicate (*n* ≥ 3), and the results are reported as mean ± standard deviation. Error bars are included in all plots to ensure data reliability and reproducibility.

### 2.5. Adsorption performance evaluation

Batch adsorption tests were systematically carried out to investigate the adsorption performance of various adsorbents, including ACLP, La_5_, La_7_, La_9_, La_15_, and non-hydrothermally treated La_9_-NH (La_9_–ACLP-NH), toward tetracycline (TC) removal. In a typical experiment, 0.03 g of each material was added to 20 mL of TC solution with a precisely prepared initial concentration of 25 mg L^−1^ at pH 6. The mixtures were agitated in a shaking incubator at 175 rpm for 90 min under ambient temperature conditions (25 ± 1 °C) to ensure sufficient contact between adsorbent and adsorbate.

Following adsorption, the suspensions were centrifuged at 4000 rpm for 10 min to separate the solid phase. The supernatants were subsequently filtered through a 0.22 µm PTFE membrane to remove any remaining particulates. The residual TC concentration was then quantified using high-performance liquid chromatography (HPLC). The calibration curve for TC determination by HPLC is presented in Fig. S2 (SI).

All experiments were performed in triplicate under identical conditions, and the reported data represent the average values.

The removal efficiency (%*R*) and adsorption capacity at time *t* (*q*_*t*_, mg g^−1^) were calculated according to the following equations:2
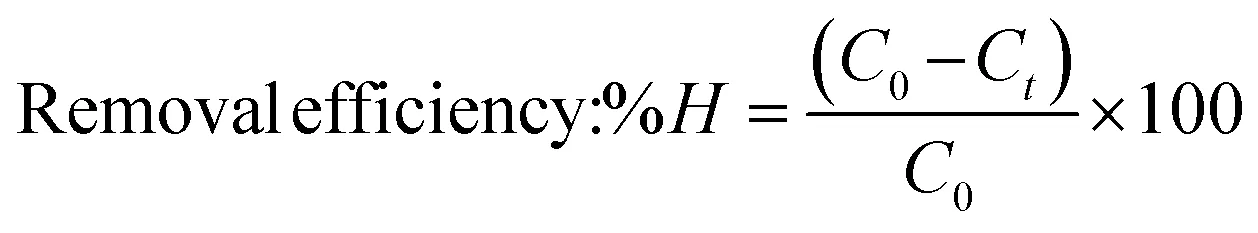
3
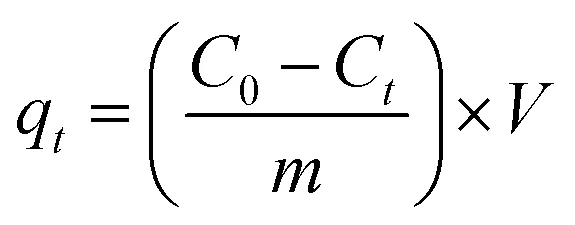
where *C*_0_ (mg L^−1^) and *C*_*t*_ (mg L^−1^) denote the initial and residual TC concentrations at time *t*, respectively; *V* (L) is the solution volume, and *m* (g) is the mass of adsorbent used.

### 2.6. Advanced modeling

#### 2.6.1 Kinetic modeling

The adsorption kinetics of tetracycline (TC) onto La_9_–ACLP were analyzed using several nonlinear kinetic models, including pseudo-first-order (PFO), pseudo-second-order (PSO), Elovich, Avrami, Weber–Morris intraparticle diffusion, and the Hameed–Daud film diffusion model. Each model was applied to the same experimental dataset to separate contributions from surface reactions and mass transfer.

PFO and PSO models were used to describe the overall adsorption rate and to estimate the rate of occupation of active sites. The Elovich equation was included to account for surface heterogeneity, while the Avrami model was used to capture possible deviations from simple kinetic behavior, particularly in systems with distributed activation energies.

To examine transport limitations, the Weber–Morris model was used to evaluate intraparticle diffusion. The Hameed–Daud model was applied to assess external mass transfer resistance across the liquid film. These two approaches allow differentiation between diffusion within pores and transport through the boundary layer.

Kinetic parameters, including rate constants (*k*_1_, *k*_2_, *k*_id_), Elovich constants (*α*, *β*), Avrami exponent (*n*), and equilibrium adsorption capacity (*q*_e_), were obtained by nonlinear regression. Experimental data were fitted directly to the original equations without linear transformation to avoid distortion over the full time range.

The Weber–Morris plots were further examined for multi-stage behavior. Deviations from linearity were used to identify changes in rate-controlling steps during adsorption. Model fitting quality was assessed using *R*^2^, *χ*^2^, RMSE, and AIC values. These metrics were used to compare model performance and to identify the dominant kinetic pathway governing TC uptake on La_9_–ACLP.

#### 2.6.2 Isotherm modeling

Equilibrium adsorption of tetracycline onto La_9_–ACLP was described using nonlinear isotherm models, including Langmuir, Freundlich, Temkin, Dubinin–Radushkevich (D–R), Sips, Toth, and Halsey equations. The selected isotherm models represent different adsorption assumptions, including monolayer adsorption, surface heterogeneity, multilayer interactions, and energetically distributed adsorption environments. All parameters were estimated by nonlinear regression using the original forms of the isotherm equations. The fitting procedure minimized the sum of squared errors (SSE) between measured and calculated adsorption capacities.

The Langmuir model provided the maximum adsorption capacity (*q*_max_) and affinity constant (*b*). Freundlich constants (*K*_F_ and *n*) were used to describe adsorption intensity and surface heterogeneity. Temkin parameters reflect adsorbate–adsorbent interactions and heat distribution. Sips and Toth models were included to account for deviations from ideal Langmuir behavior at higher concentrations. The D–R model was applied to estimate the mean adsorption energy (*E*), which helps distinguish between physical and chemical interactions. The dimensionless separation factor (*R*_L_) derived from the Langmuir model was used to evaluate adsorption favorability over the studied concentration range. Model performance was evaluated using *R*^2^, *χ*^2^, RMSE, and AIC. These criteria were used to compare the suitability of different models across the full concentration range.

#### 2.6.3 Adsorption thermodynamics

Thermodynamic parameters were determined to describe the temperature dependence of TC adsorption on La_9_–ACLP. Equilibrium experiments were conducted at different temperatures (*e.g.*, 298, 313, and 323 K) under identical conditions, including adsorbent dosage, pH, initial concentration, and contact time.

At equilibrium, the distribution coefficient (*K*_d_, L g^−1^) was calculated as:

4 *K*_d_ = *q*_e_/*C*_e_

where *q*_e_ (mg g^−1^) is the equilibrium adsorption capacity and *C*_e_ (mg L^−1^) is the equilibrium concentration of TC in solution.

The standard Gibbs free energy change (Δ*G*°, kJ mol^−1^) was determined using:

5 Δ*G*° = −*RT* ln *K*_d_

where *R* is the universal gas constant (8.314 J mol^−1^ K^−1^) and *T* is the absolute temperature (K).

The standard enthalpy (Δ*H*°, kJ mol^−1^) and entropy (Δ*S*°, J mol^−1^ K^−1^) changes were calculated from the Van't Hoff equation:

6 ln *K*_d_ = −(Δ*H*°/*RT*) + (Δ*S*°/*R*)

The values of Δ*H*° and Δ*S*° were obtained from the slope and intercept of the linear plot of ln *K*_d_*versus* 1/*T*, respectively.

*K*_d_ was treated as an apparent equilibrium parameter under dilute conditions. All measurements were conducted in triplicate, and averaged values were used for calculation.

### 2.7. Statistical analysis

All experimental data and Figures were processed and performed using Origin Pro 2019 (USA). Statistical significance and model fitting accuracy were evaluated using *R*^2^, RMSE, *χ*^2^, and AIC parameters. Error analysis was based on triplicate measurements, and uncertainties are reported as standard deviations.

## 3. Results and discussion

### 3.1. Structural and surface characteristics governing TC adsorption onto La_9_–ACLP

Lanthanum incorporation modified the pore structure and surface properties of the activated carbon. Accordingly, physicochemical characterization focused on pore accessibility, surface functional groups, and electrostatic interactions, which are closely related to TC adsorption capacity and kinetics.

#### 3.1.1 Structural identification by XRD

**Fig. 2:**
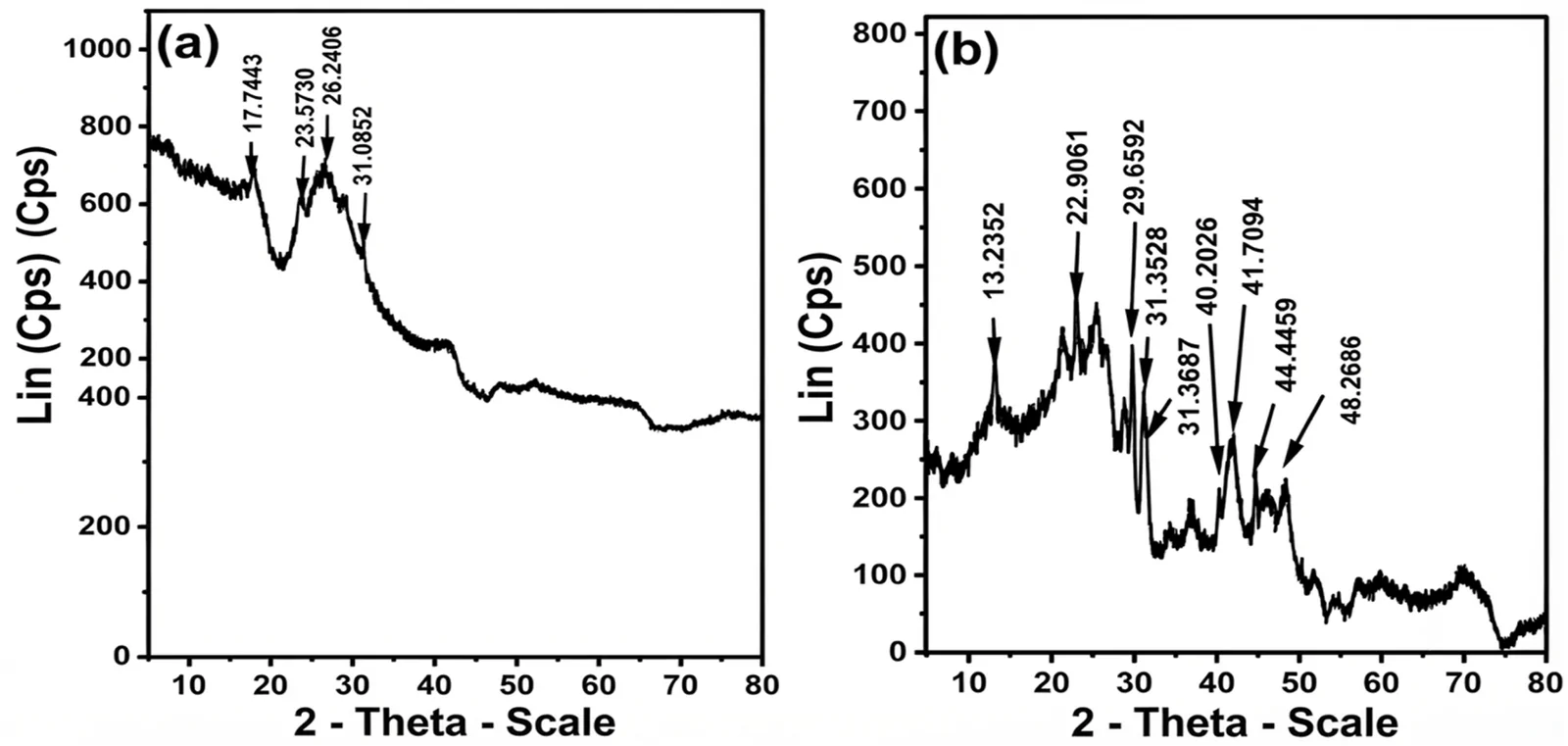
XRD patterns of (a) ACLP and (b) La_9_–ACLP.

The XRD patterns of ACLP and La_9_–ACLP are shown in Fig. 2. The ACLP sample (Fig. 2a) displays broad diffraction features centered at 2*θ* ≈ 17.7°, 23.57°, and 26.24°, which are typical of amorphous carbon with a low degree of graphitic ordering.^1^ The broadness of these reflections reflects a turbostratic structure in which graphene-like layers are randomly stacked without long-range crystallographic coherence.^2^ Such structural disorder is commonly observed in biomass-derived carbons and is associated with abundant defect sites and chemically accessible surface regions.^29,30^

After lanthanum incorporation, additional reflections appear in the pattern of La_9_–ACLP (Fig. 2b) at 2*θ* ≈ 29.66°, 31.37°, 40.20°, 41.71°, 44.45°, and 48.27°, corresponding to the (101), (110), (102), (111), (200), and (201) planes of hexagonal La_2_O_3_ (JCPDS No. 05-0602). The relatively weak intensity and slight peak broadening suggest that lanthanum oxide is present as finely dispersed nanocrystalline domains rather than large crystallites. Such dispersion may increase interfacial contact between oxide and carbon while maintaining accessible surface area.^31,32^ The persistence of broad carbon-related bands indicates that the turbostratic framework of ACLP remains largely intact after modification. Minor reflections at 2*θ* ≈ 13.2° and 22.9° are attributed to La(OH)_3_ or La_2_O_2_CO_3_ species formed through partial hydration and carbonation of La_2_O_3_ upon exposure to moisture and CO_2_.^33^ These features suggested that lanthanum species were successfully incorporated without disrupting the defective carbon structure.

#### 3.1.2 Morphology and elemental distribution (SEM, TEM, EDS)

**Fig. 3:**
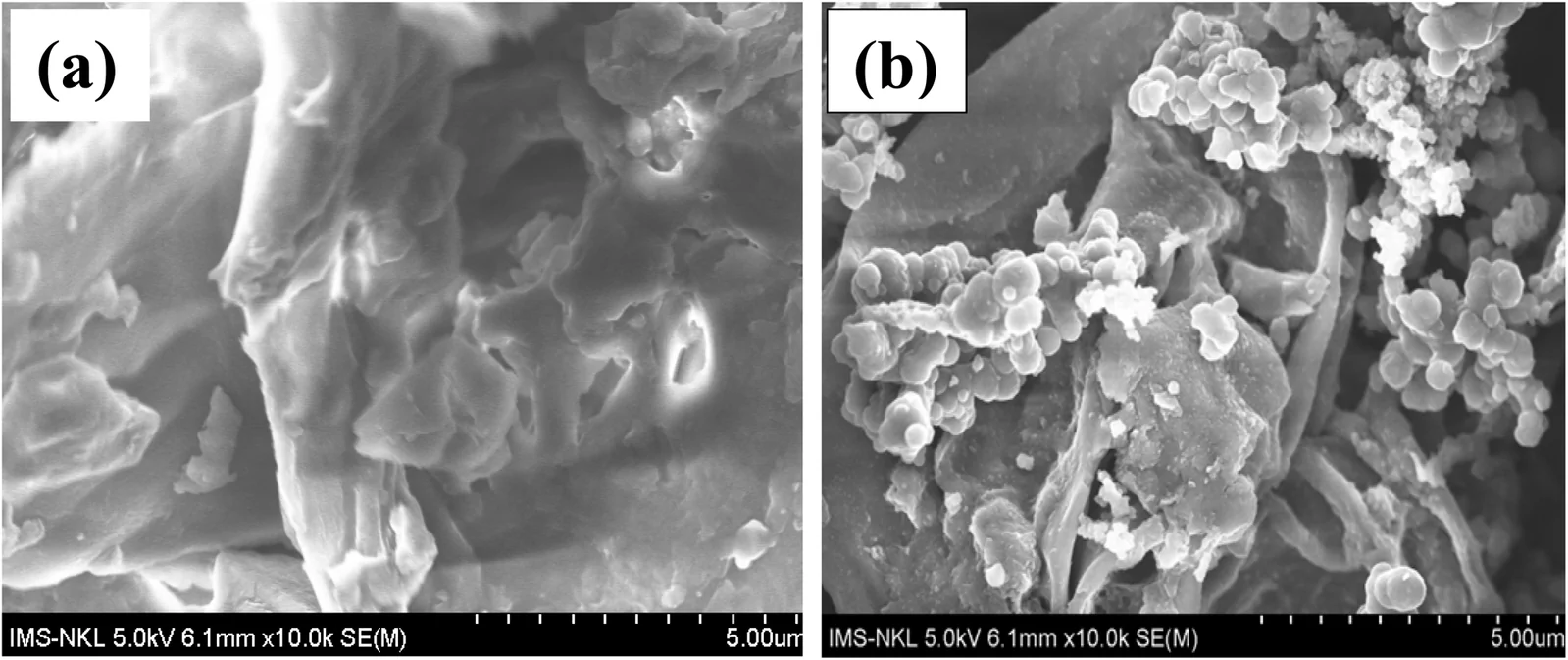
SEM images of (a) ACLP and (b) La_9_–ACLP.

SEM images (Fig. 3) show that pristine ACLP consists of irregularly stacked carbon sheets forming aggregated domains. After modification, numerous bright nanoparticles are distributed across the carbon surface, with apparent sizes of approximately 20–30 nm. These bright regions correspond to lanthanum-containing phases with higher electron density than the carbon matrix. Their widespread presence suggests deposition across multiple surface sites rather than localized growth.

**Fig. 4:**
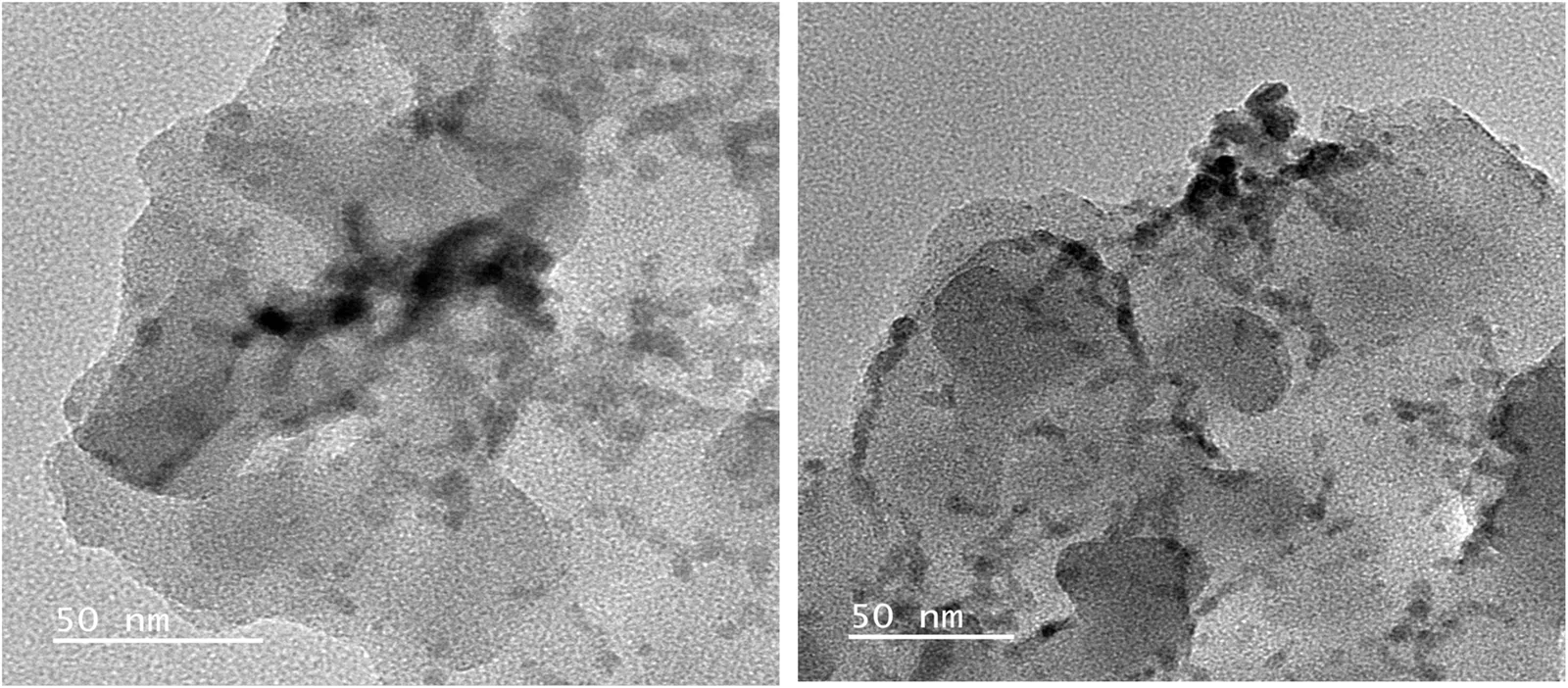
TEM image of La_9_–ACLP.

TEM analysis (Fig. 4) provides a clearer view of the microstructure. The carbon support appears as thin, low-contrast sheets, while darker regions correspond to La_2_O_3_ nanoparticles. Particle sizes observed by TEM fall within the range of 5–15 nm, which is smaller than those observed in SEM images. This difference is attributed to aggregation during drying or sample preparation for SEM. The observations suggest that La_2_O_3_ initially forms as small primary particles that subsequently assemble into larger clusters. At this scale, a higher fraction of surface atoms may remain exposed, which may increase the number of accessible interfacial sites. Such behavior is consistent with size-dependent reactivity in supported metal oxide systems.^31,32^

**Fig. 5:**
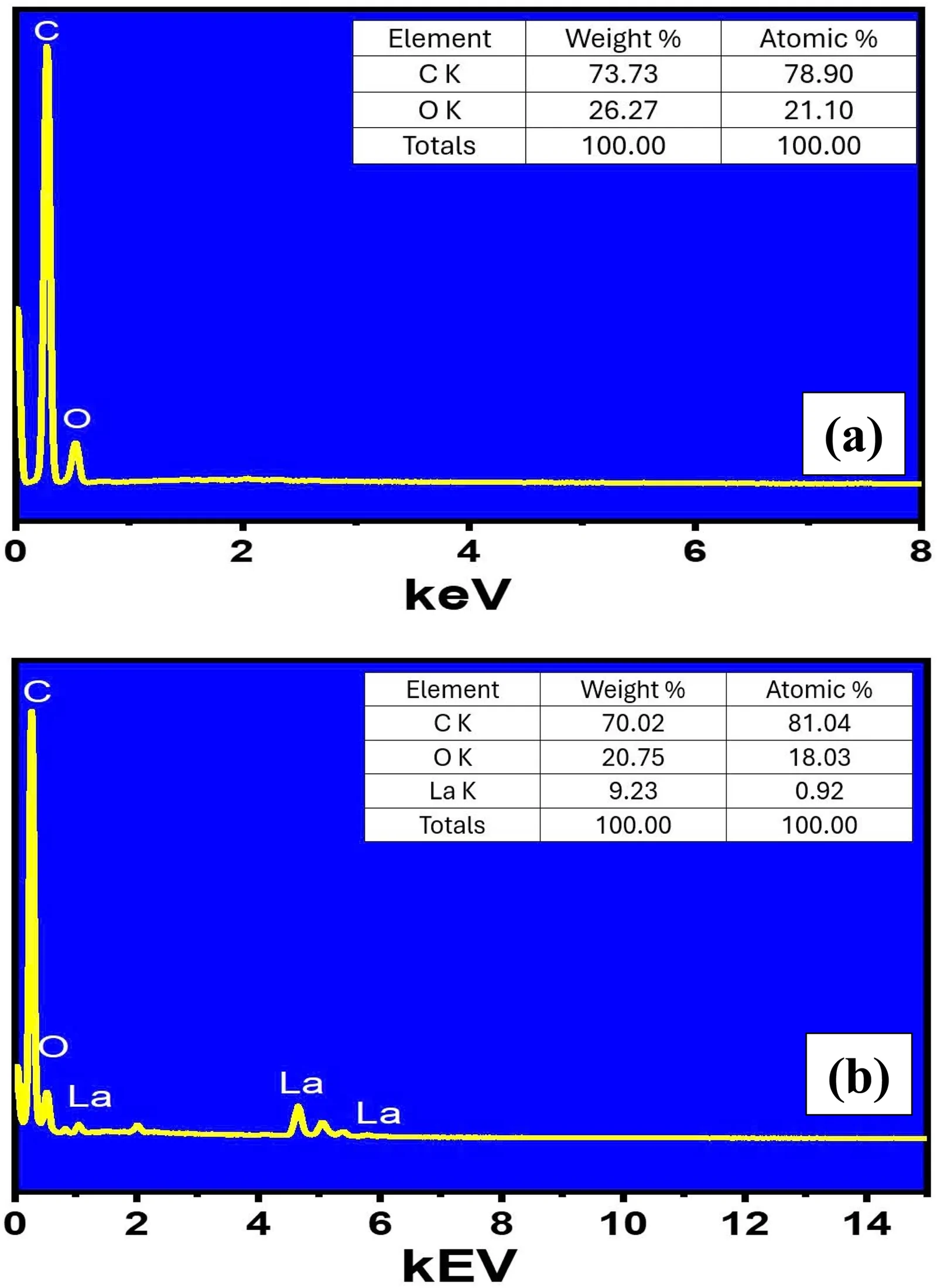
EDS spectra of (a) ACLP and (b) La_9_–ACLP.

The EDS results of ACLP and La_9_–ACLP are presented in Fig. 5. Pristine ACLP is dominated by C (73.73 wt%; 78.90 at%) and O (26.27 wt%; 21.10 at%), consistent with oxygen-functionalized biomass-derived carbon (Fig. 5a). After modification, La_9_–ACLP shows the emergence of La (9.23 wt%; 0.92 at%), accompanied by slight decreases in C (70.02 wt%) and O (20.75 wt%), reflecting the introduction of lanthanum species onto the carbon framework (Fig. 5b). The absence of concentrated La-rich regions suggests a relatively uniform dispersion, in agreement with the XRD and TEM observations.

#### 3.1.3 Surface chemistry and interaction mechanisms (FT-IR)

**Fig. 6:**
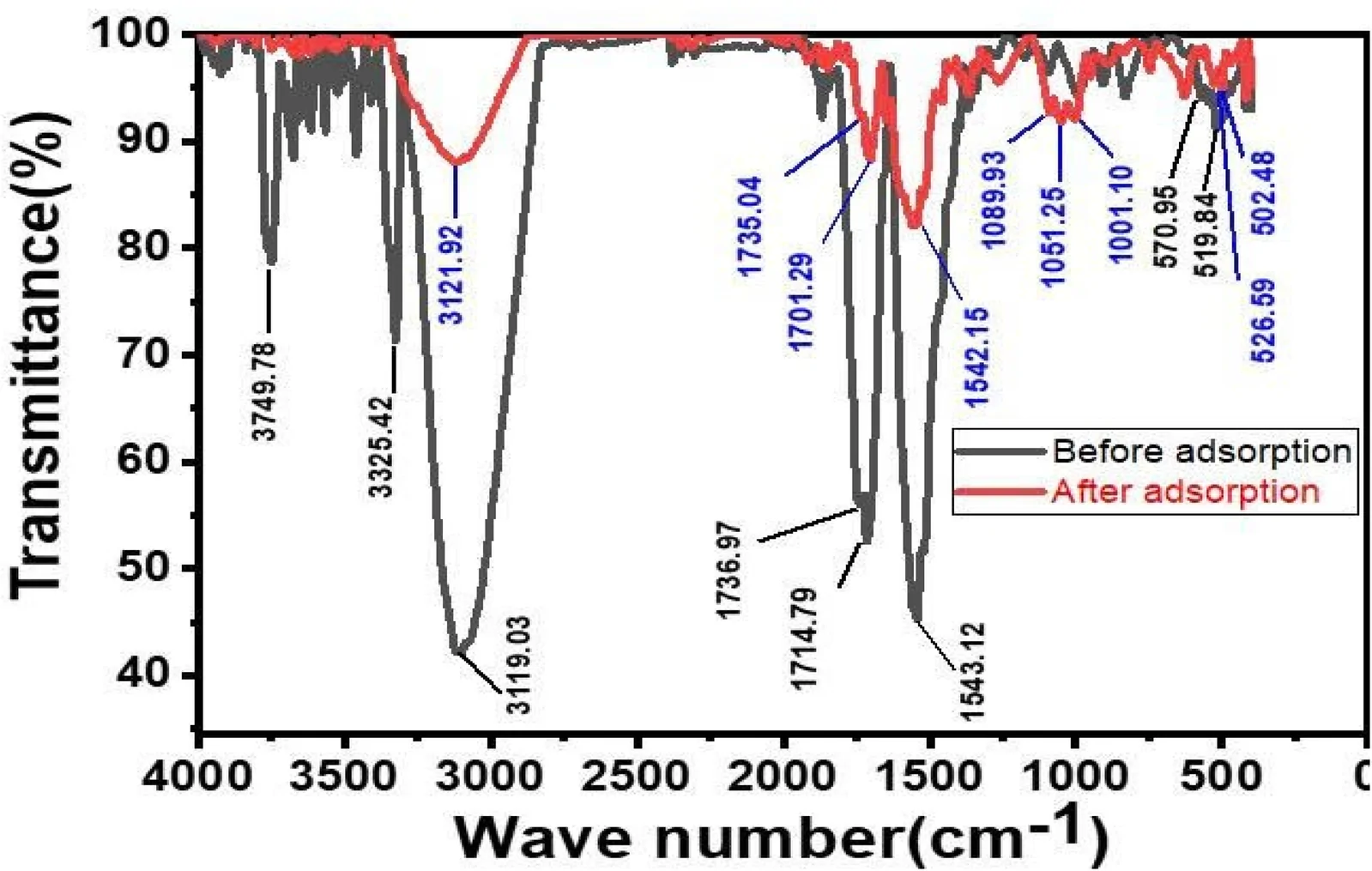
FT-IR spectra of La_9_–ACLP before and after TC adsorption.

The FT-IR spectrum of La_9_–ACLP (Fig. 6 and Table 1) shows characteristic bands associated with –OH, –OH/N–H, C=O, aromatic C=C, and C–O/C–N functional groups, indicating that oxygen-containing groups derived from the precursor remain after modification. Bands in the region of 560–520 cm^−1^ are assigned to La–O vibrations, confirming the presence of lanthanum oxide species.^34,35^

**Table 1:** FT-IR band assignments before and after adsorption TC

Wavenumber (cm^−1^)	Functional group	Before adsorption	After adsorption
3749.78	–OH	Sharp band, medium intensity	Disappeared
3325.42	–OH/N–H	Sharp band, medium intensity	Disappeared
3119.03–3121.92	C–H	Broad band, strong intensity	Decreased intensity with a slight shift
1736.97–1714.79	C=O	Strong intensity	Decreased intensity with a slight shift
1543.12–1542.15	C=C (aromatic)	Strong intensity	Decreased intensity with a slight shift
1089.93–1001.10	C–O/C–N	Weak intensity	Increased intensity with a slight shift
570.84–526.59	La–O	Weak intensity	Slight shift

After adsorption of tetracycline, several spectral changes are observed. Bands associated with –OH/N–H decrease in intensity or disappear, while the C=O and aromatic C=C bands shift slightly and show reduced intensity. Signals in the C–O/C–N region become more pronounced, and small shifts are observed in the La–O vibration region. These changes point to the involvement of multiple interaction pathways. The decrease in hydroxyl-related bands suggests hydrogen bonding between surface groups and polar functional groups of tetracycline. Changes in the aromatic region indicate interactions between conjugated carbon domains and the aromatic rings of tetracycline, consistent with π–π interactions.^36,37^

Changes observed in the La–O vibration region point toward the participation of lanthanum-containing surface domains during tetracycline adsorption. Incorporation of La_2_O_3_ does more than introduce additional surface species; it creates La–O environments that are chemically distinct from the surrounding carbon framework. Lanthanum-containing oxide domains are commonly regarded as Lewis acidic sites capable of interacting with oxygen-donor ligands through surface complexation and coordination-related interactions.^22,38^ In the present system, electron-rich functionalities of tetracycline, particularly phenolic, amide, and diketone groups, represent plausible interaction sites. Within this context, the spectral changes observed after adsorption suggest the involvement of La-containing interfacial domains in tetracycline uptake, although the specific nature of these interactions cannot be resolved from FTIR data alone.^36^

At the same time, caution is warranted when interpreting FTIR evidence. Band shifts alone cannot resolve the exact nature of the interaction or establish a specific coordination geometry between tetracycline and lanthanum species. The observed changes are more appropriately interpreted as indirect evidence of localized interactions involving lanthanum-associated adsorption sites rather than definitive proof of La–TC complex formation. Direct identification of such complexes would require complementary surface-sensitive techniques, including XPS, EXAFS, or related spectroscopic approaches capable of probing the local coordination environment in greater detail.^39,40^

The adsorption surface is unlikely to be chemically uniform. Oxygen-containing functional groups inherited from the carbon matrix coexist with lanthanum-containing domains, generating adsorption sites with different chemical characteristics and affinities toward tetracycline species. Under these conditions, adsorption is more plausibly described as the combined outcome of several interaction pathways, including hydrogen bonding, π-related interactions, surface complexation, and localized lanthanum-associated interactions, rather than a single dominant adsorption mechanism.^36^ The FTIR results are consistent with such a multi-interaction adsorption environment, although a more detailed description of the interfacial structure would require advanced spectroscopic characterization.^39,40^

#### 3.1.4 Surface chemical states revealed by XPS

**Fig. 7:**
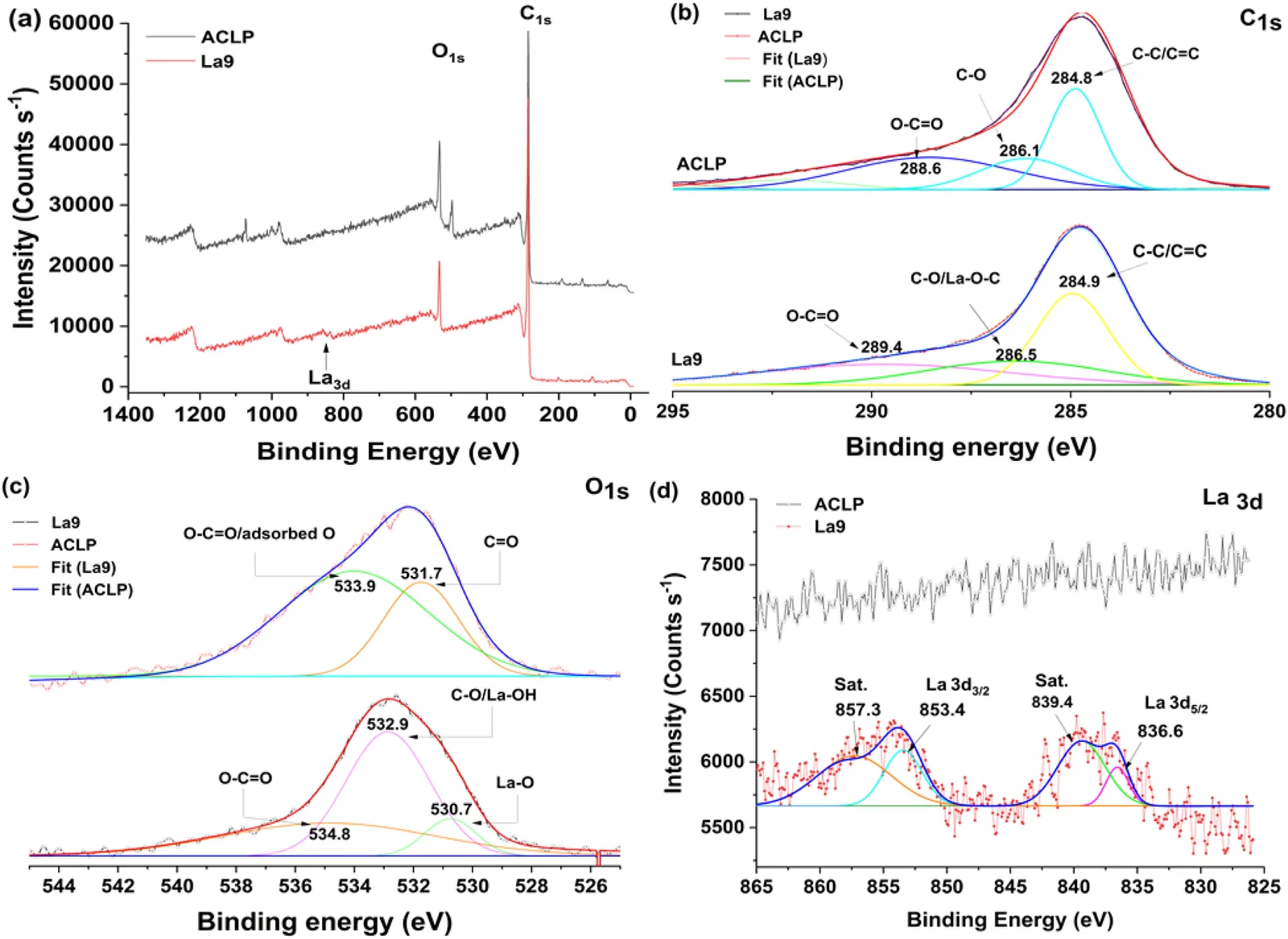
X-ray photoelectron spectroscopy (XPS) characterization of pristine activated carbon (ACLP) and lanthanum-modified activated carbon (La_9_–ACLP or La_9_).

XPS analysis was performed to examine the surface chemical composition and oxidation states of the pristine activated carbon (ACLP) and the lanthanum-modified sample (La_9_–ACLP) (Fig. 7).

The survey spectra show the dominant C 1s and O 1s signals for both materials, whereas distinct La 3d peaks appear only after lanthanum modification, confirming the successful incorporation of lanthanum onto the outer surface of the activated carbon. Together with the XRD and SEM–EDS analyses, these results indicate that lanthanum is present not only within the composite but also at chemically accessible surface sites. The high-resolution C 1s spectrum (Fig. 7b) can be deconvoluted into three components centred at approximately 284.8–284.9, 286.0–286.1, and 288.6–289.4 eV, corresponding to C–C/C=C, C–O, and O–C=O/C=O species, respectively. Only minor binding-energy shifts are observed following lanthanum incorporation, suggesting that the carbon framework remains largely preserved, while localized changes occur in the chemical environment surrounding oxygen-containing surface functionalities.

The O 1s spectrum (Fig. 7c) consists of three contributions centred near 529.2, 530.7, and 531.7 eV, assigned to lattice oxygen in La–O, hydroxylated oxygen (La–OH/C–O), and carboxyl/carbonyl oxygen (O–C=O), respectively. The appearance of the La–O component is consistent with the presence of lanthanum oxide species on the carbon surface, whereas oxygen-containing functional groups inherited from the biomass precursor remain evident after lanthanum modification.

The high-resolution La 3d spectrum (Fig. 7d) exhibits the characteristic La 3d_5/2_ and La 3d_3_/_2_ doublet centred at approximately 833.4 and 850.4 eV, together with well-resolved shake-up satellites at 836.6 and 853.7 eV, which are characteristic of trivalent lanthanum (La^3+^) in La_2_O_3_. No additional components attributable to metallic or reduced lanthanum species are detected within the resolution of the present measurements. The absence of reduced lanthanum species further suggests that hydrothermal deposition followed by calcination stabilizes lanthanum predominantly in its thermodynamically favoured oxide form. These observations are consistent with the XRD results, indicating that lanthanum is predominantly present as oxidized La_2_O_3_ on the activated carbon surface.

Overall, the XPS results establish the initial surface chemical state of the lanthanum-modified activated carbon by revealing the presence of surface-exposed La_2_O_3_ together with preserved carbon and oxygen-containing surface functionalities. These observations complement the structural information obtained from XRD, SEM-EDS, and BET, whereas their significance for tetracycline adsorption is discussed comprehensively in Section 3.7.

#### 3.1.5 Surface area and pore architecture revealed by N_2_ adsorption (BET analysis)

**Fig. 8:**
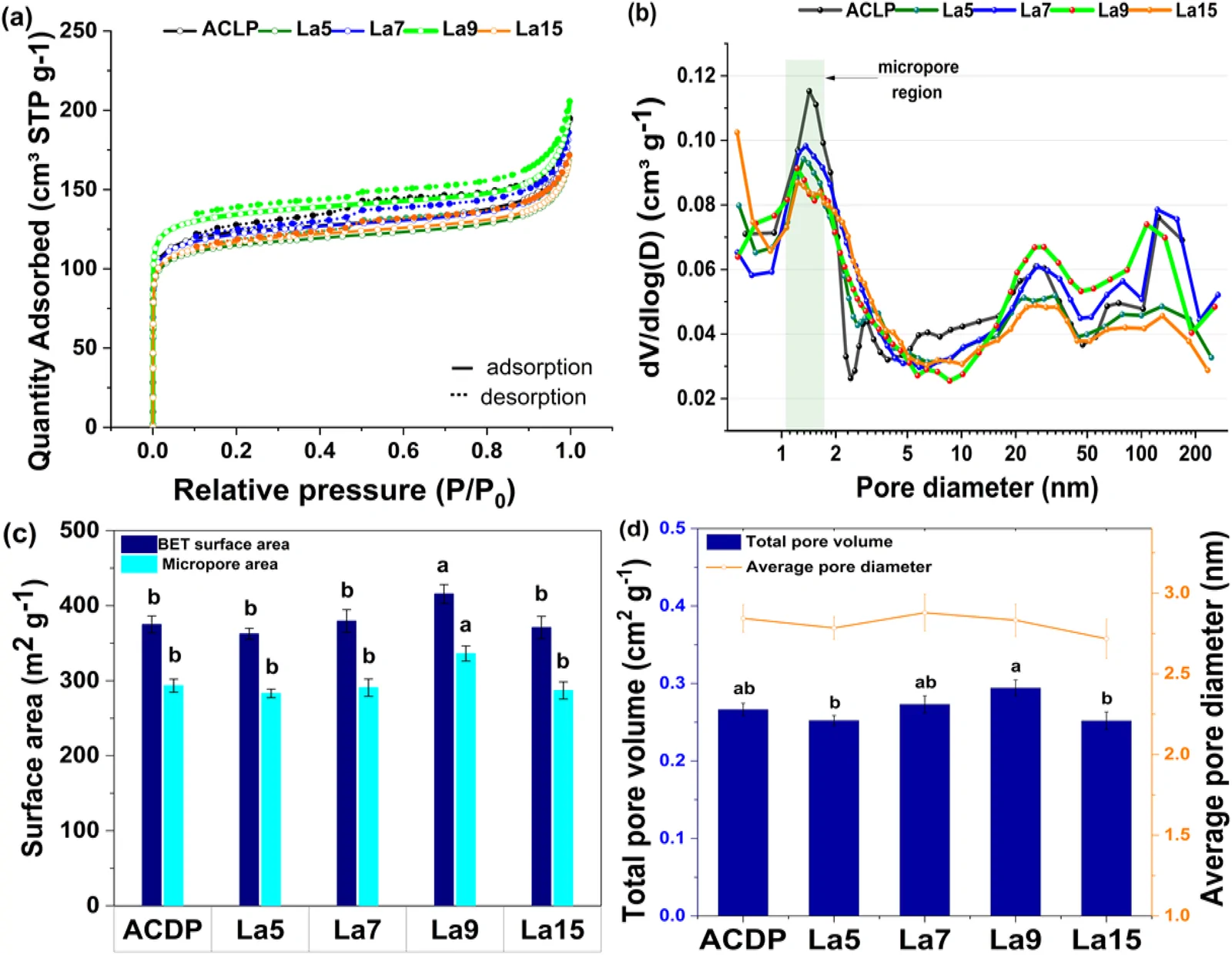
Textural characterization of pristine activated carbon (ACLP) and lanthanum-modified activated carbons (La_5_, La_7_, La_9_, and La_15_) by N_2_ adsorption–desorption analysis. (a) N_2_ adsorption–desorption isotherms. (b) BJH pore-size distributions. (c) BET specific surface area and micropore surface area. (d) Total pore volume and average pore diameter. Error bars represent the standard deviation (*n* = 3), and different lowercase letters indicate statistically significant differences (*P* < 0.05).

The evolution of the porous structure with increasing lanthanum loading is characterized by non-monotonic changes in the textural properties of the activated carbons (Fig. 8). Comparison of ACLP, La_5_, La_7_, La_9_, and La_15_ reveals how lanthanum incorporation modifies the pore structure while largely preserving the hierarchical pore architecture.

The N_2_ adsorption–desorption isotherms (Fig. 8a) of all samples exhibit type IV behaviour with well-developed hysteresis loops at intermediate and high relative pressures, characteristic of mesoporous carbon materials according to the IUPAC classification. The overall isotherm profiles remain largely unchanged after lanthanum incorporation, suggesting that hydrothermal deposition followed by calcination does not fundamentally disrupt the mesoporous architecture generated during H_3_PO_4_ activation. Instead, lanthanum incorporation appears to primarily influence the existing pore structure while preserving the overall pore architecture.

The quantitative textural parameters derived from the adsorption isotherms (Fig. 8c and d) further support this interpretation. Relative to pristine ACLP, lanthanum incorporation is accompanied by reductions in BET specific surface area together with moderate variations in total pore volume and average pore diameter. These changes are consistent with partial occupation of accessible pore surfaces by deposited La_2_O_3_ nanoparticles rather than collapse of the carbon framework. Notably, none of the measured textural parameters varies monotonically with increasing nominal lanthanum loading, indicating that the evolution of the measured pore characteristics does not scale proportionally with lanthanum loading.

The pore-size distributions (Fig. 8b) provide complementary structural information. All samples retain pronounced microporous features centred at approximately 1–2 nm, together with a broad mesoporous population extending from approximately 20 to more than 100 nm, indicating that the hierarchical pore architecture established during H_3_PO_4_ activation is maintained after lanthanum modification. Mesoporous features remain evident throughout the series, further supporting preservation of the hierarchical pore architecture following lanthanum incorporation, whereas the gradual reduction in micropore accessibility at higher nominal lanthanum loadings is consistent with localized occupation of pore entrances by deposited La_2_O_3_ nanoparticles rather than structural collapse of the pore framework.

Collectively, the adsorption isotherms (Fig. 8a), pore-size distributions (Fig. 8b), and quantitative textural parameters (Fig. 8c and d) indicate that lanthanum incorporation modifies the pore structure while preserving the hierarchical porous architecture of the biomass-derived activated carbon. These observations establish the structural framework for interpreting the adsorption behaviour discussed in Section 3.7. The mechanistic significance of these structural characteristics is discussed together with the complementary adsorption and surface-characterization results presented in the following sections.

#### 3.1.6 Thermal stability (TGA analysis)

**Fig. 9:**
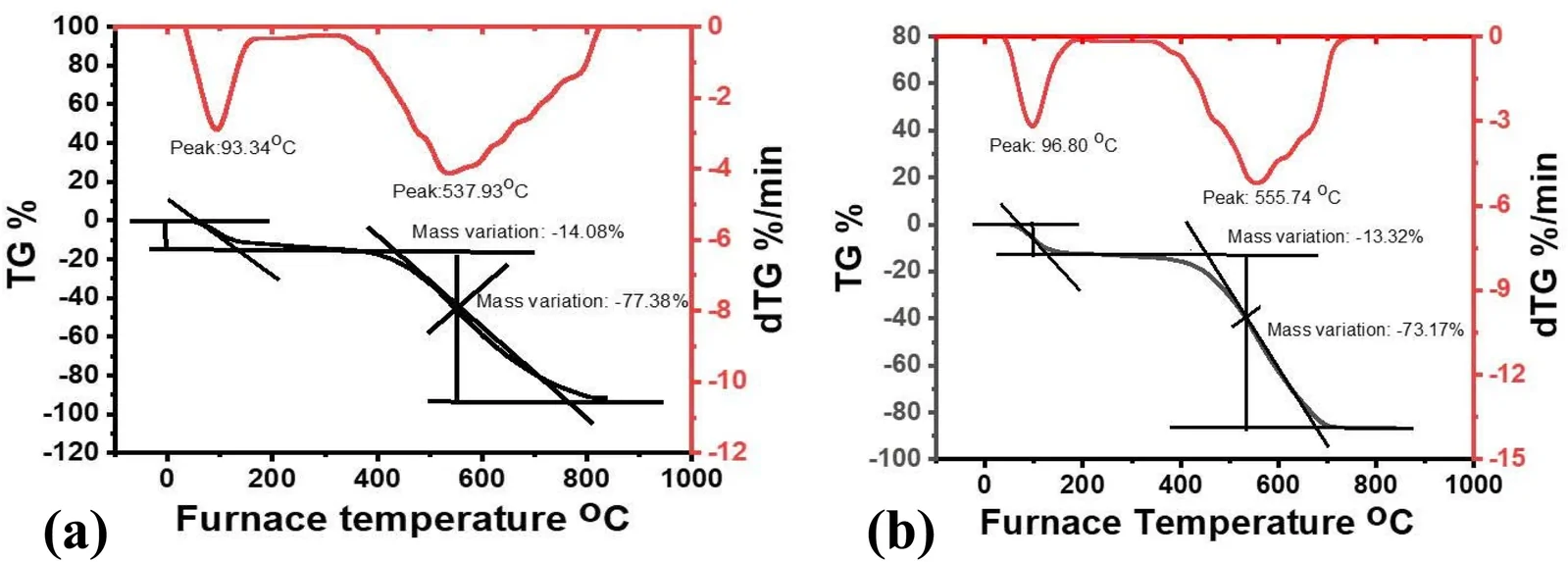
TG–DTG curves of ACLP (a) and La_9_–ACLP (b).

TG–DTG curves (Fig. 9) display a multistep decomposition profile typical of lignocellulosic-derived carbon materials. Initial weight loss below 150 °C corresponds to the removal of physically adsorbed water. The region between 150 and 400 °C is associated with decomposition of residual oxygen-containing groups,^17^ while the range from 400 to 700 °C corresponds to degradation of the carbon framework.^41^

Compared with ACLP, La_9_–ACLP exhibits reduced mass loss at lower temperatures and a shift of the main DTG peak from 537.93 to 554.74 °C. The total weight loss is also lower. These changes indicate improved thermal stability after lanthanum incorporation. The presence of lanthanum species stabilizes reactive surface groups and strengthens interactions between oxide and carbon. At temperatures above 700 °C, higher residual mass is observed due to the presence of thermally stable lanthanum-containing phases and increased fixed-carbon content. This enhanced stability is relevant for regeneration processes involving thermal treatment.

#### 3.1.7 Surface charge properties (pH_pzc_)

**Fig. 10:**
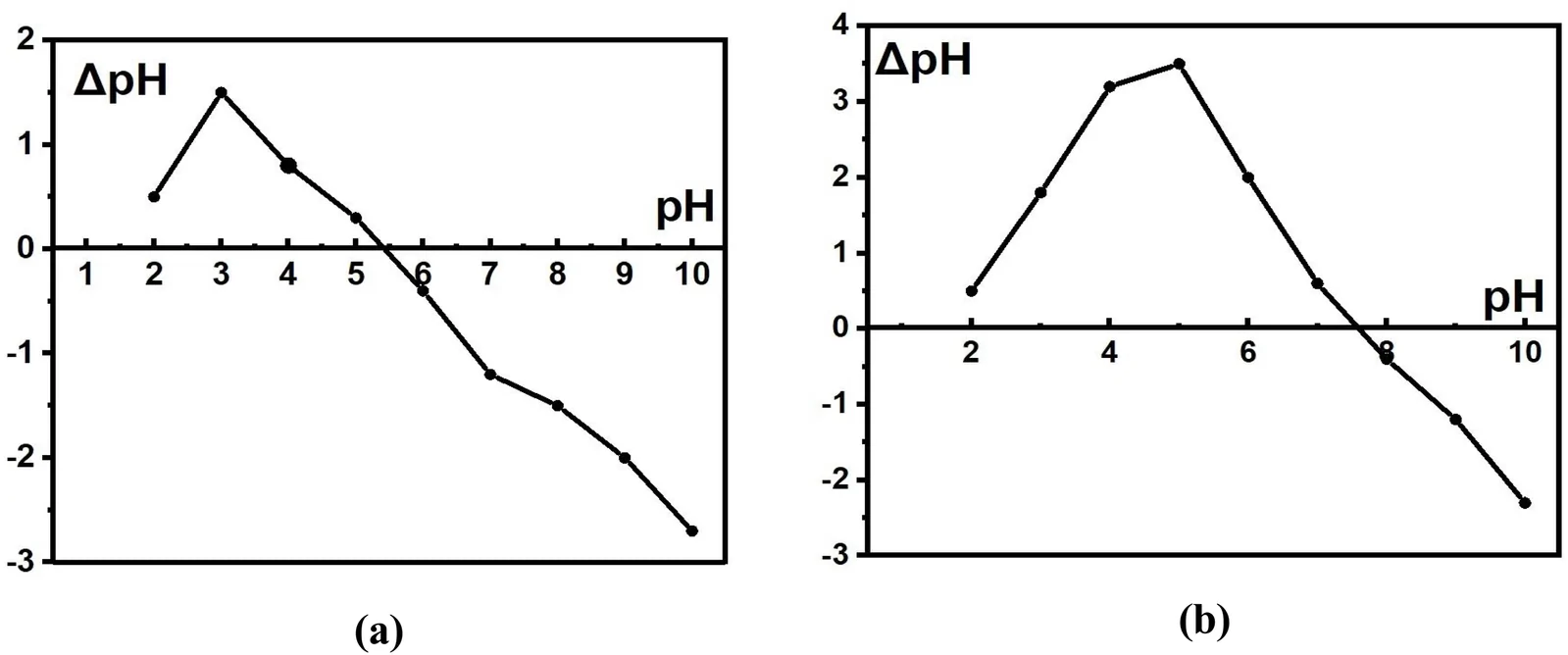
Determination of pH_pzc_ for (a) ACLP and (b) La_9_–ACLP determined at room temperature (25 °C).

The pH_p_zc increased from 5.5 for ACLP to 7.6 after lanthanum incorporation (Fig. 10), reflecting a substantial shift in the acid–base character of the carbon surface. The observed increase suggests that lanthanum incorporation modifies the protonation behaviour and interfacial charge characteristics of the carbon surface.^42^ These observations are consistent with the incorporation of lanthanum oxide species identified by XRD and XPS, together with the preservation of oxygen-containing surface functionalities revealed by FTIR.

Because the surface charge of carbon materials depends on the relationship between the solution pH and pH_p_zc, the observed shift indicates that lanthanum modification alters the surface charge behaviour over a broad pH range. Since tetracycline exists in different protonation states depending on solution pH, changes in pH_p_zc provide an important physicochemical descriptor of the adsorbent surface under different solution conditions. The implications of these surface charge properties for tetracycline adsorption are discussed in conjunction with the complementary structural, surface, and adsorption results presented in Section 3.7.

### 3.2. Evaluation of key factors influencing tetracycline adsorption onto La_9_–ACLP

The adsorption study was organized progressively to examine operational adsorption behavior, adsorption kinetics, equilibrium characteristics, thermodynamic responses, and interaction mechanisms of TC uptake onto La_9_–ACLP. All adsorption experiments were performed in triplicate under identical conditions. Reported values correspond to mean results to ensure reproducibility.

#### 3.2.1 Effect of pH

**Fig. 11:**
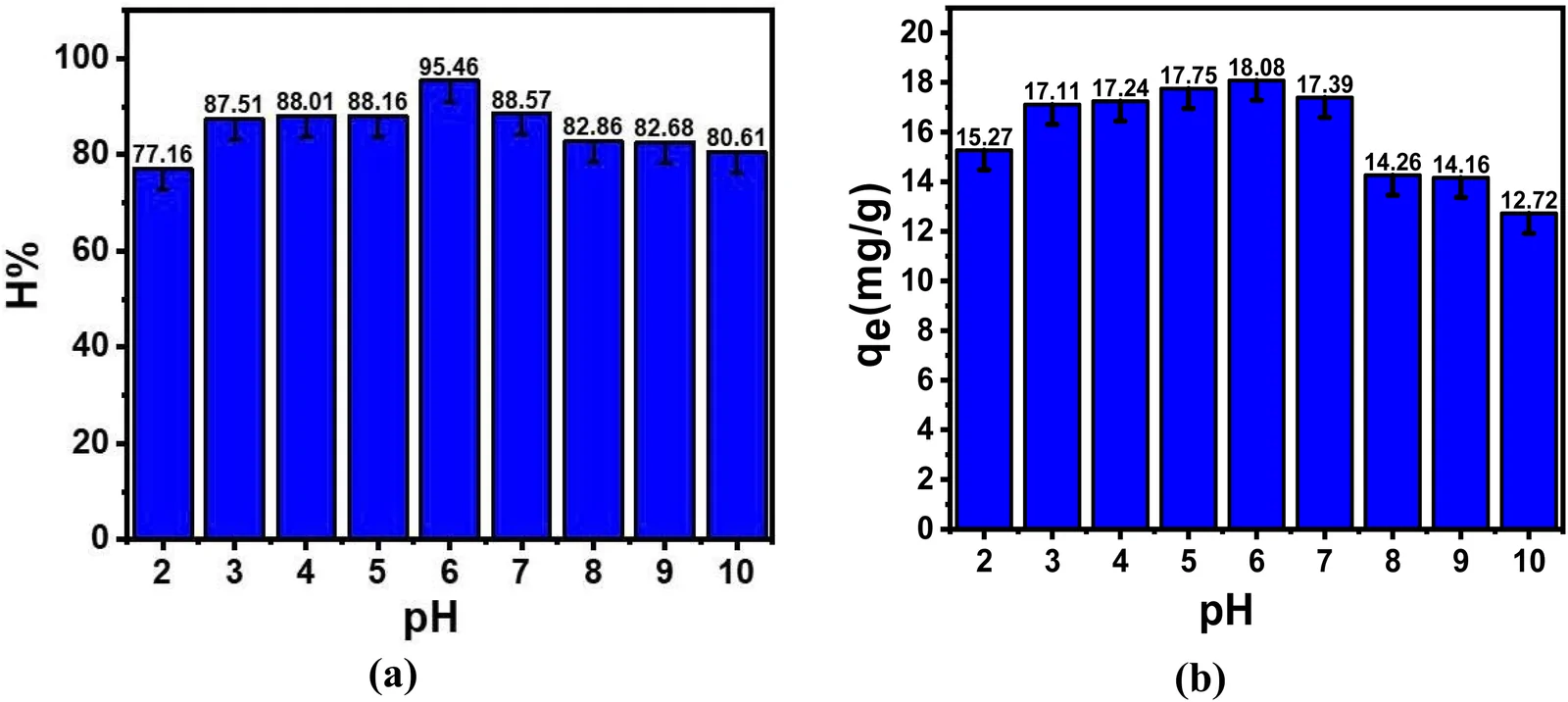
Effect of solution pH on tetracycline (TC) adsorption onto La_9_–ACLP. Experimental conditions: *C*_0_ = 25 mg L^−1^, adsorbent dosage = 0.03 g, contact time = 90 min, and *T* = 298 K. Data are presented as mean ± standard deviation from triplicate experiments (*n* = 3), and error bars represent standard deviation.

The adsorption behavior of La_9_–ACLP exhibited a pronounced dependence on solution pH, with maximum TC uptake observed near pH ≈ 6 and progressively lower adsorption under strongly acidic or alkaline conditions (Fig. 11). This trend reflects the coupled influence of TC aqueous speciation and the evolving surface-charge characteristics of the adsorbent.

Under strongly acidic conditions (pH ≈ 2), protonation of surface functional groups likely reduces the availability of electron-donating adsorption sites. TC is also present mainly in cationic form at low pH, which may weaken interactions with positively charged surface regions and lower adsorption uptake.^43^

The highest adsorption capacity was observed near neutral pH (pH ≈ 6). Under these conditions, TC exists predominantly as a zwitterionic species, while electrostatic effects become less important. π–π interactions, hydrogen bonding, and interactions associated with lanthanum-containing surface sites may all contribute to adsorption at near-neutral pH.^27,44^

At alkaline pH (pH > 7–8), TC gradually shifts toward anionic forms while the adsorbent surface becomes increasingly deprotonated. Electrostatic repulsion becomes more pronounced, and OH^−^ ions may also compete for adsorption sites, leading to lower TC uptake.^44^

Adsorption was less favorable under strongly acidic and alkaline conditions, whereas the highest uptake occurred near neutral pH.

#### 3.2.2 Effect of contact time

**Fig. 12:**
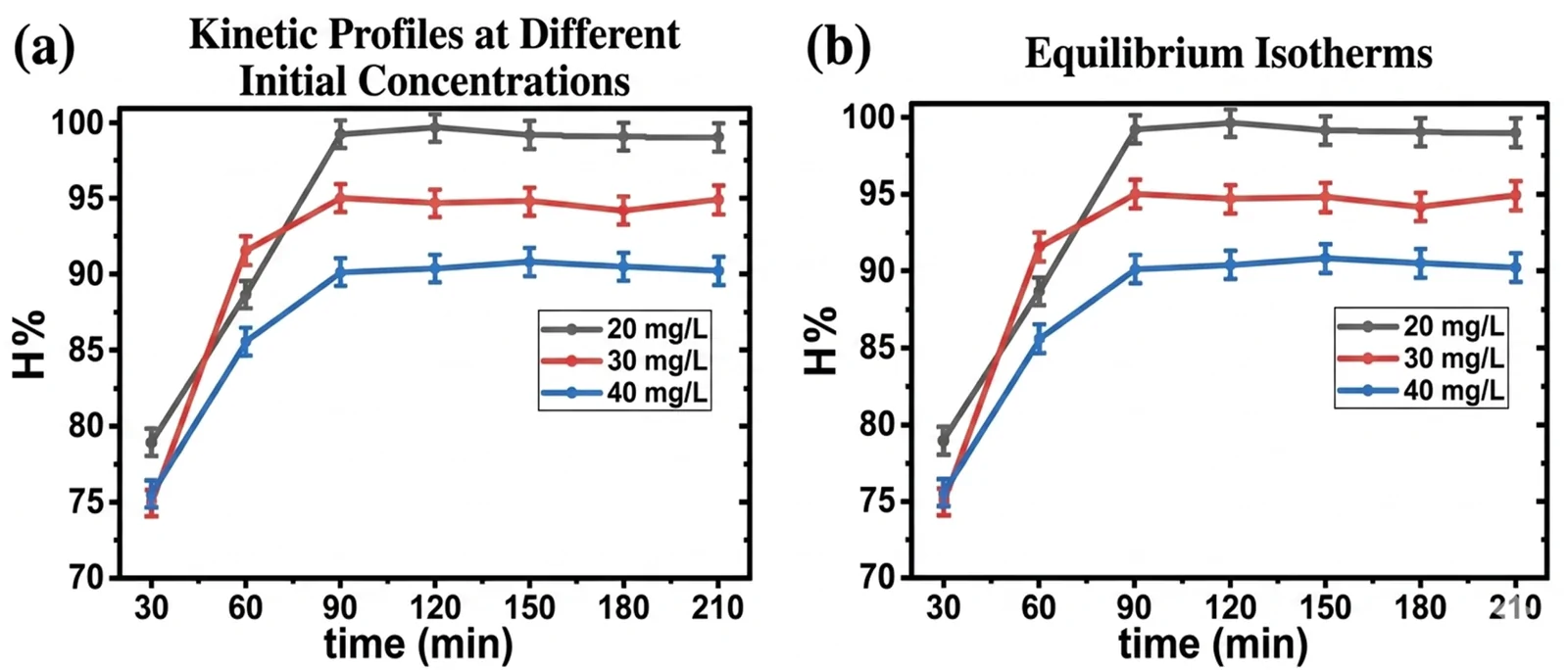
Dependence of (a) removal efficiency and (b) adsorption capacity on contact time at initial TC concentrations of 20, 30, and 40 mg L^−1^. Experimental conditions: adsorbent dosage = 0.03 g, pH = 6.0, and *T* = 298 K. Data are presented as mean ± standard deviation from triplicate experiments (*n* = 3), and error bars represent standard deviation.

TC adsorption increased rapidly at the beginning of the process and gradually reached equilibrium after approximately 90 min (Fig. 12).

During the first 60 min, TC uptake increased sharply because a large number of adsorption sites were still available and the concentration gradient between the solution and adsorbent surface remained high. The adsorption rate then decreased with increasing contact time. Similar behavior has been widely reported for porous carbon adsorbents and is generally attributed to progressive occupation of adsorption sites together with slower intraparticle diffusion at later adsorption stages.^45^

A slight decrease in removal efficiency was observed at higher initial TC concentrations (30–50 mg L^−1^). This effect was likely related to stronger competition for favorable adsorption sites at higher surface loading. An equilibrium contact time of approximately 90 min was selected for the subsequent adsorption and kinetic experiments.^46^

The contact–time profiles presented here were subsequently analyzed using kinetic models, discussed in Section 3.3.

#### 3.2.3 Effect of adsorbent dosage

**Fig. 13:**
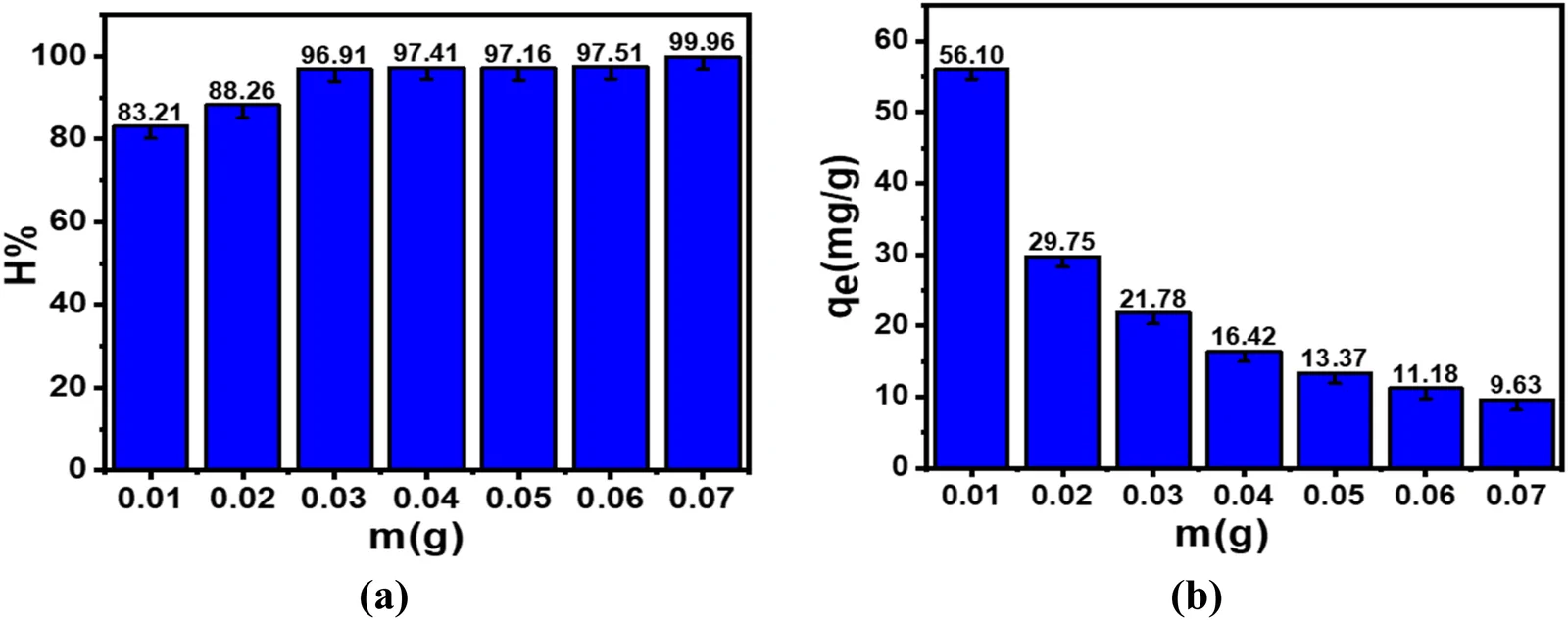
Dependence of (a) removal efficiency and (b) adsorption capacity on adsorbent dosage during TC adsorption onto La_9_–ACLP. Experimental conditions: *C*_0_ ≈ 30 mg L^−1^, pH = 6.0, contact time = 90 min, and *T* = 298 K. Data are presented as mean ± standard deviation from triplicate experiments (*n* = 3), and error bars represent standard deviation.

Increasing the adsorbent dosage from 0.01 to 0.03 g increased TC removal efficiency from 83.22% to 96.91% (Fig. 13). This behavior was mainly due to the larger number of available adsorption sites and the greater available surface area at higher adsorbent loading.

Further increases in dosage above 0.03 g produced only minor improvements in removal efficiency, which gradually approached a plateau (∼99.96%). At higher dosage, the amount of dissolved TC became relatively low compared with the available adsorption capacity.

In contrast, the equilibrium adsorption capacity (*q*_e_) decreased from 21.78 to 9.63 mg g^−1^ as adsorbent dosage increased. Similar trends have been reported in systems with excess adsorbent, where a larger fraction of adsorption sites remains unused at higher solid-to-solution ratios. Partial particle aggregation at elevated dosage may also reduce effective surface accessibility and decrease site-utilization efficiency.^47^

An adsorbent dosage of 0.03 g per 25 mL was selected for the subsequent experiments because it provided high TC removal without excessive adsorbent consumption.

#### 3.2.4 Effect of temperature

**Fig. 14:**
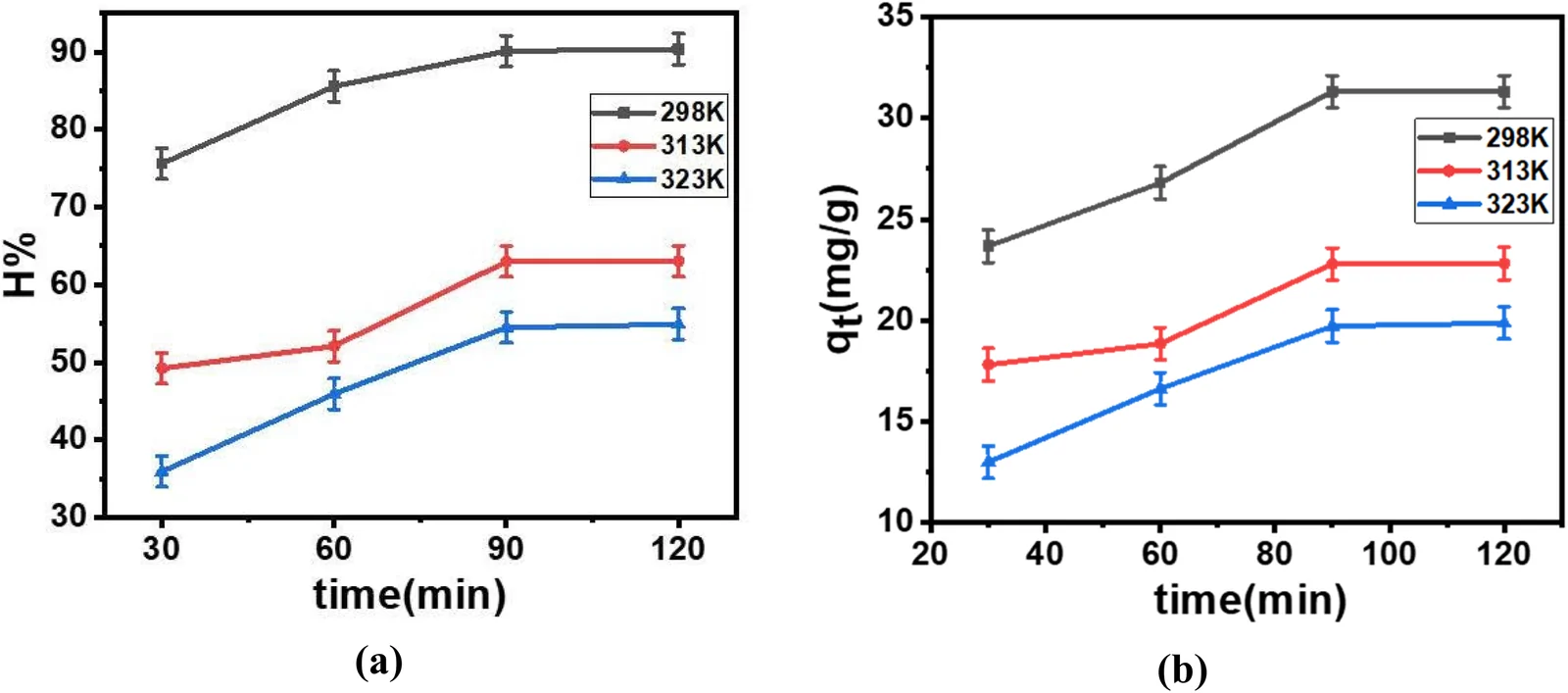
Dependence of (a) removal efficiency and (b) adsorption capacity on temperature. Data are presented as mean ± standard deviation from triplicate experiments (*n* = 3), and error bars represent standard deviation.

Increasing the temperature from 298 to 323 K gradually decreased TC adsorption efficiency (Fig. 14). Such behavior is commonly observed in predominantly exothermic adsorption systems. At elevated temperature, TC adsorption on La_9_–ACLP became less favorable, resulting in lower adsorption performance. Higher molecular mobility together with weaker adsorbate–surface interactions at elevated temperature may reduce adsorption uptake on porous carbon materials.^48^

The temperature dependence of TC adsorption was further examined through thermodynamic analysis, as discussed in Section 3.5.

#### 3.2.5 Effect of initial TC concentration

**Fig. 15:**
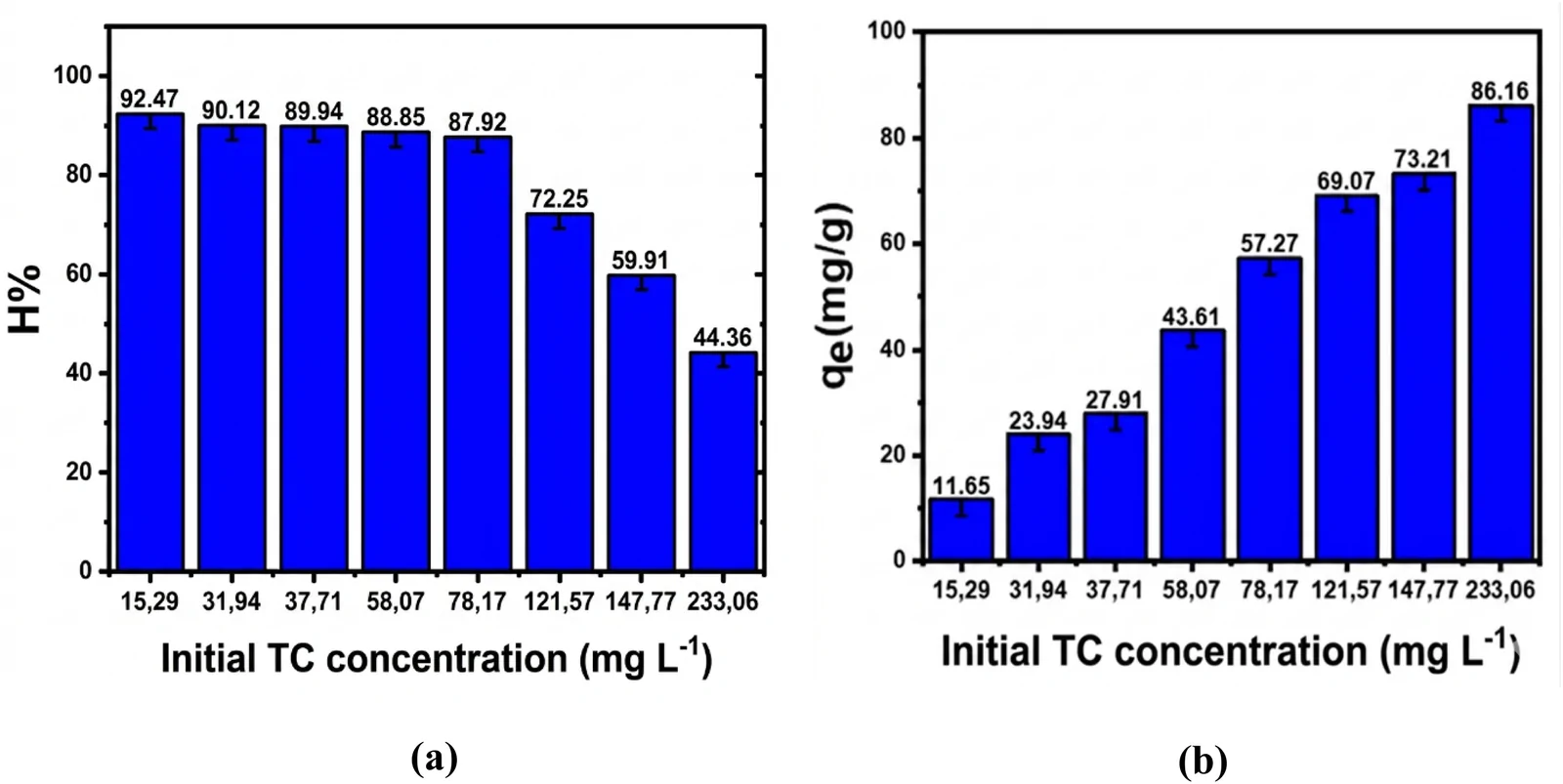
Dependence of (a) removal efficiency and (b) adsorption capacity on initial TC concentration (*C*_0_). Data are presented as mean ± standard deviation from triplicate experiments (*n* = 3), and error bars represent standard deviation.

At lower initial TC concentrations (15.29–78.17 mg L^−1^), TC removal efficiency remained relatively high (92.47–87.92%), while the equilibrium adsorption capacity increased from 11.66 to 57.27 mg g^−1^ (Fig. 15). Under these conditions, the concentration gradient between the bulk solution and the adsorbent surface remained sufficiently large to support efficient mass transfer, while many adsorption sites were still available for TC uptake. At higher initial TC concentrations (>100 mg L^−1^), removal efficiency gradually decreased from 72% to 44.36%, whereas adsorption capacity continued to increase and reached 86.16 mg g^−1^.

The lower removal efficiency at elevated TC concentrations is likely related to progressive occupation of favorable adsorption sites and reduced site availability at higher surface loading. At the same time, the stronger concentration gradient at higher TC concentration continued to promote adsorption uptake, resulting in higher equilibrium adsorption capacity.

The effect of initial TC concentration on adsorption behavior was further examined using equilibrium isotherm analysis, as discussed in Section 3.4.

### 3.3. Adsorption kinetics

TC adsorption onto La_9_–ACLP occurs within a heterogeneous porous framework influenced by surface interactions, pore diffusion, and mass-transfer effects. Under these conditions, no single kinetic model can adequately represent all adsorption behavior across the investigated concentration range. Multiple kinetic models were therefore evaluated to compare adsorption-rate behavior and diffusion-related contributions during TC uptake.

The adsorption kinetics of tetracycline (TC) onto La_9_–ACLP were analyzed using a series of nonlinear models, including pseudo-first-order (PFO), pseudo-second-order (PSO), Elovich, Avrami, Weber–Morris intraparticle diffusion, and the Hameed–Daud film diffusion model. Each model was applied to the same dataset to separate the contributions of surface reactions and transport processes within the porous structure.

PFO and PSO describe the evolution of site occupation over time. The Elovich and Avrami models capture heterogeneity in surface energy and variations in adsorption pathways. The Weber–Morris and film diffusion models were used to distinguish intraparticle diffusion from external mass transfer. Together, these models provide a more resolved description of the system than any individual approach.

**Fig. 16:**
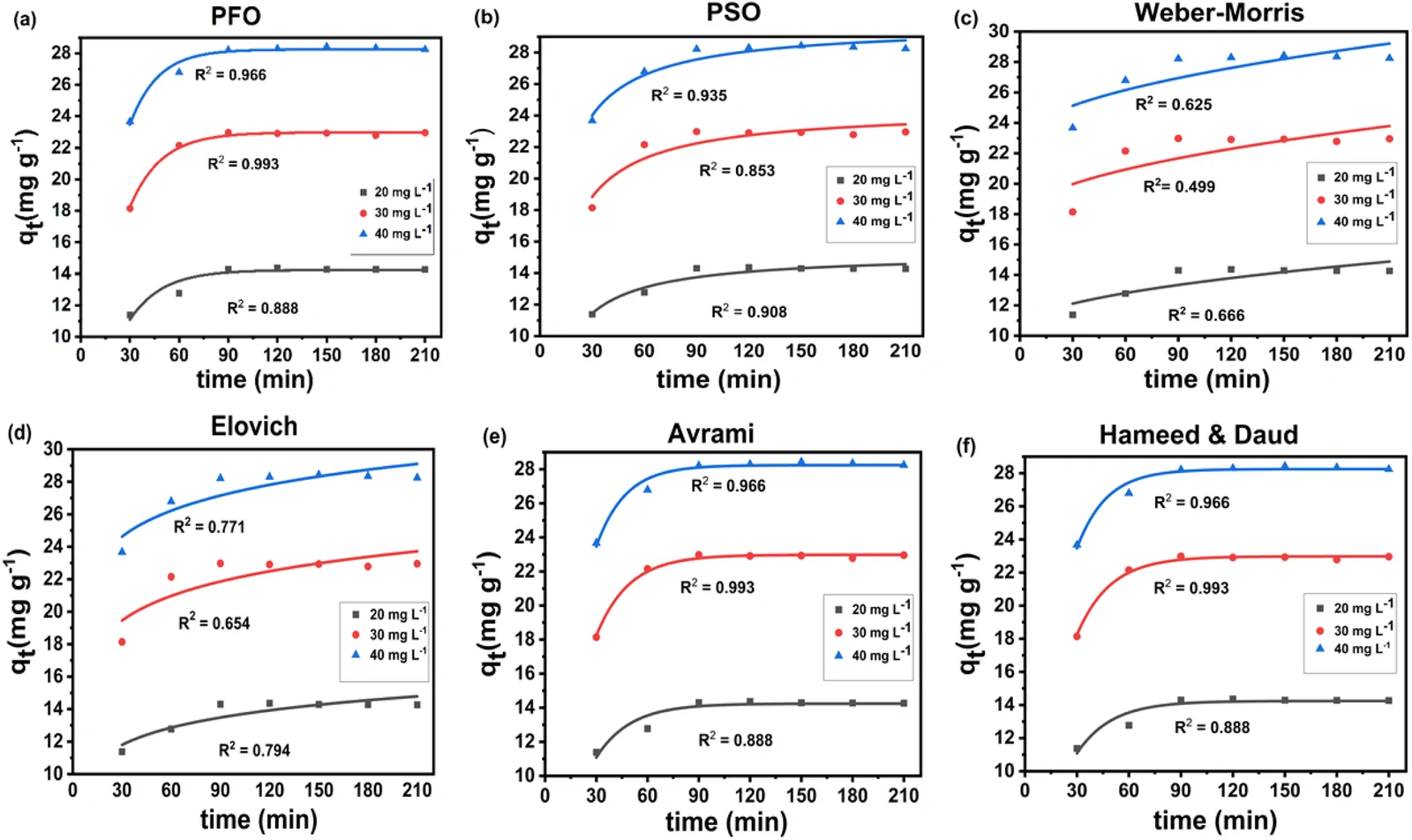
Kinetic fits of *q*_e_ as a function of time (*i*) using (a) pseudo-first-order (PFO), (b) pseudo-second-order (PSO), (c) Weber–Morris intraparticle diffusion, (d) Elovich, (e) Avrami, and (f) film diffusion (Hameed–Daud) models.

Nonlinear fits of *q*_*t*_*versus* time at different initial concentrations are presented in Fig. 16, with the corresponding parameters listed in Tables 2 and 3. The fitting behavior changed systematically with the initial TC concentration, suggesting that the relative contributions of surface interactions and mass–transfer processes varied across different adsorption conditions. At the same time, mechanistic interpretation of the kinetic analysis should remain cautious because several models produced statistically comparable fits within overlapping uncertainty ranges. For heterogeneous porous adsorbents such as La_9_–ACLP, kinetic models are therefore better treated as empirical descriptions of adsorption behavior rather than direct evidence for distinct adsorption mechanisms.

**Table 2:** Fitted kinetic constants obtained from nonlinear kinetic models at different initial TC concentrations (*C*_0_)

*C*_0_ (mg L^−1^)	Model	Parameter	Value
20	PFO	*q*_e_ (mg g^−1^)	14.242
		*k*_1_ (min^−1^)	0.0502
	PSO	*q*_e_ (mg g^−1^)	15.260
		*k*_2_ (L mg^−1^ min^−1^)	0.0065
	Elovich	*α* (mg g^−1^ min^−1^)	109.92
		*β* (g mg^−1^)	0.6504
	Avrami	*k* (min^−1^)	0.0502
		*q*_e_ (mg g^−1^)	14.242
	Weber–Morris	*k*_i_ *d* (mg g^−1^ min^−1/2^)	0.3069
		*C* (mg g^−1^)	10.429
	Film diffusion	*k*_f_ (min^−1^)	0.0502
		*q*_e_ (mg g^−1^)	14.242
30	PFO	*q*_e_ (mg g^−1^)	22.976
		*k*_1_ (min^−1^)	0.0526
	PSO	*q*_e_ (mg g^−1^)	24.433
		*k*_2_ (L mg^−1^ min^−1^)	0.00460
	Elovich	*α* (mg g^−1^ min^−1^)	523.90
		*β* (g mg^−1^)	0.4562
	Avrami	*k* (min^−1^)	0.0525
		*q*_e_ (mg g^−1^)	22.976
	Weber–Morris	*k*_i_ *d* (mg g^−1^ min^−1/2^)	0.4237
		*C* (mg g^−1^)	17.651
	Film diffusion	*k*_f_ (min^−1^)	0.0525
		*q*_e_ (mg g^−1^)	22.976
40	PFO	*q*_e_ (mg g^−1^)	28.245
		*k*_1_ (min^−1^)	0.0592
	PSO	*q*_e_ (mg g^−1^)	29.755
		*k*_2_ (L mg^−1^ min^−1^)	0.0046
	Elovich	*α* (mg g^−1^ min^−1^)	3423.3
		*β* (g mg^−1^)	0.4347
	Avrami	*k* (min^−1^)	0.0592
		*q*_e_ (mg g^−1^)	28.245
	Weber–Morris	*k*_i_ *d* (mg g^−1^ min^−1^/^2^)	0.4533
		*C* (mg g^−1^)	22.644
	Film diffusion	*k*_f_ (min^−1^)	0.0592
		*q*_e_ (mg g^−1^)	28.245

**Table 3:** Kinetic model parameters, statistical indicators, and goodness-of-fit ranking at different initial concentrations (*C*_0_)

*C*_0_ (mg L^−1^)	Model	*R* ^2^	RMSE (mg g^−1^)	*χ* ^2^	AIC	ΔAIC
20	PFO	0.9065	0.327	0.056	−11.63	13.37
	PSO	0.9235	0.296	0.044	−13.03	11.97
	Elovich	0.8284	0.443	0.101	−7.38	17.63
	Avrami	0.9065	0.327	0.056	−11.63	13.37
	Weber–Morris	0.7220	0.564	0.167	−4.00	21.00
	Film diffusion	0.9065	0.327	0.057	−11.63	13.38
30	PFO	0.9941	0.126	0.005	−25.00	0
	PSO	0.8771	0.577	0.109	−3.69	21.31
	Elovich	0.7120	0.883	0.259	2.26	27.35
	Avrami	0.9941	0.126	0.005	−25.00	0
	Weber–Morris	0.5823	1.064	0.377	4.87	29.87
	Film diffusion	0.9941	0.126	0.005	−25.01	0
40	PFO	0.9718	0.272	0.019	−14.24	10.76
	PSO	0.9460	0.376	0.036	−9.67	15.33
	Elovich	0.7002	0.707	0.130	−0.84	24.16
	Avrami	0.9718	0.271	0.019	−14.24	10.77
	Weber–Morris	0.6877	0.906	0.216	2.61	27.62
	Film diffusion	0.9718	0.272	0.019	−14.24	10.77

At *C*_0_ = 20 mg L^−1^, the PSO model showed the closest agreement with the experimental data, with *R*^2^ = 0.9235, RMSE = 0.296, and *χ*^2^ = 0.044, together with a lower AIC value than the other tested models. Under these conditions, adsorption kinetics appeared to be influenced more strongly by surface-related interactions. Similar behavior has been reported previously for TC adsorption on modified carbon materials in dilute systems.^49^

At higher TC concentrations (30–40 mg L^−1^), the fitting preference shifted toward the PFO, Avrami, and film-diffusion models. These models produced higher *R*^2^ values (≈0.97–0.99) together with lower RMSE and *χ*^2^ values. The lowest AIC value was obtained at *C*_0_ = 30 mg L^−1^ (−25), where ΔAIC = 0, indicating the strongest statistical support among the tested models.

At lower TC concentrations, adsorption kinetics were more consistent with PSO-type behavior, suggesting a stronger contribution from surface interactions when adsorption sites remained relatively accessible.^50^ As TC concentration increased, diffusion-influenced models, including PFO- and film-diffusion-type descriptions, showed progressively better agreement with the experimental kinetic profiles.^51^ This trend does not necessarily indicate a distinct mechanistic transition. More likely, it reflects gradual changes in the relative contributions of interfacial transport, pore diffusion, and site accessibility as surface coverage increased.

Table 2 summarizes the kinetic constants obtained from nonlinear fitting, while comparative fitting quality and model adequacy are presented separately in Table 3.

The Weber–Morris model gives lower *R*^2^ values (0.58–0.72) and noticeable deviations across all concentrations. The plots are not linear over the entire time range, indicating that intraparticle diffusion alone does not control the overall rate. Such behavior is characteristic of multi-step adsorption processes involving both transport and surface interactions.^52^

In contrast, the film diffusion model shows closer agreement at intermediate and high concentrations, supporting the role of external mass transfer, particularly when concentration gradients are steep at the initial stage.

The Elovich model produces weaker fits (*R*^2^ = 0.70–0.83) and larger deviations than the other models. Although often associated with heterogeneous surfaces, its limited performance here suggests that the adsorption kinetics cannot be described solely by a heterogeneous chemisorption framework. Similar observations have been reported for TC adsorption on modified biochar systems.^53^

The kinetic analysis suggests that TC adsorption on La_9_–ACLP involved coupled contributions from external mass transfer, intraparticle diffusion, and surface-related interactions, with their relative importance changing with TC concentration. Because several kinetic models showed similarly good agreement with the experimental data, the present results do not support identification of a single dominant rate-controlling step.

Instead, the adsorption process is better described as a combined transport and surface-interaction system in which multiple diffusion- and interface-related processes operate simultaneously throughout adsorption.^50^

### 3.4. Adsorption isotherm analysis

Equilibrium adsorption behavior was first evaluated experimentally using the qe–Ce relationship before applying nonlinear isotherm models to interpret adsorption-site heterogeneity and adsorption-capacity characteristics.

Equilibrium interactions between tetracycline (TC) and La_9_–ACLP were examined through non-linear isotherm modeling. This approach provides a quantitative basis for evaluating surface affinity, apparent capacity, and the distribution of adsorption energies across the material surface.^54^ Rather than relying on a single equation, multiple models were fitted in parallel to reduce selection bias and to better capture adsorption behavior over the full concentration range.

**Fig. 17:**
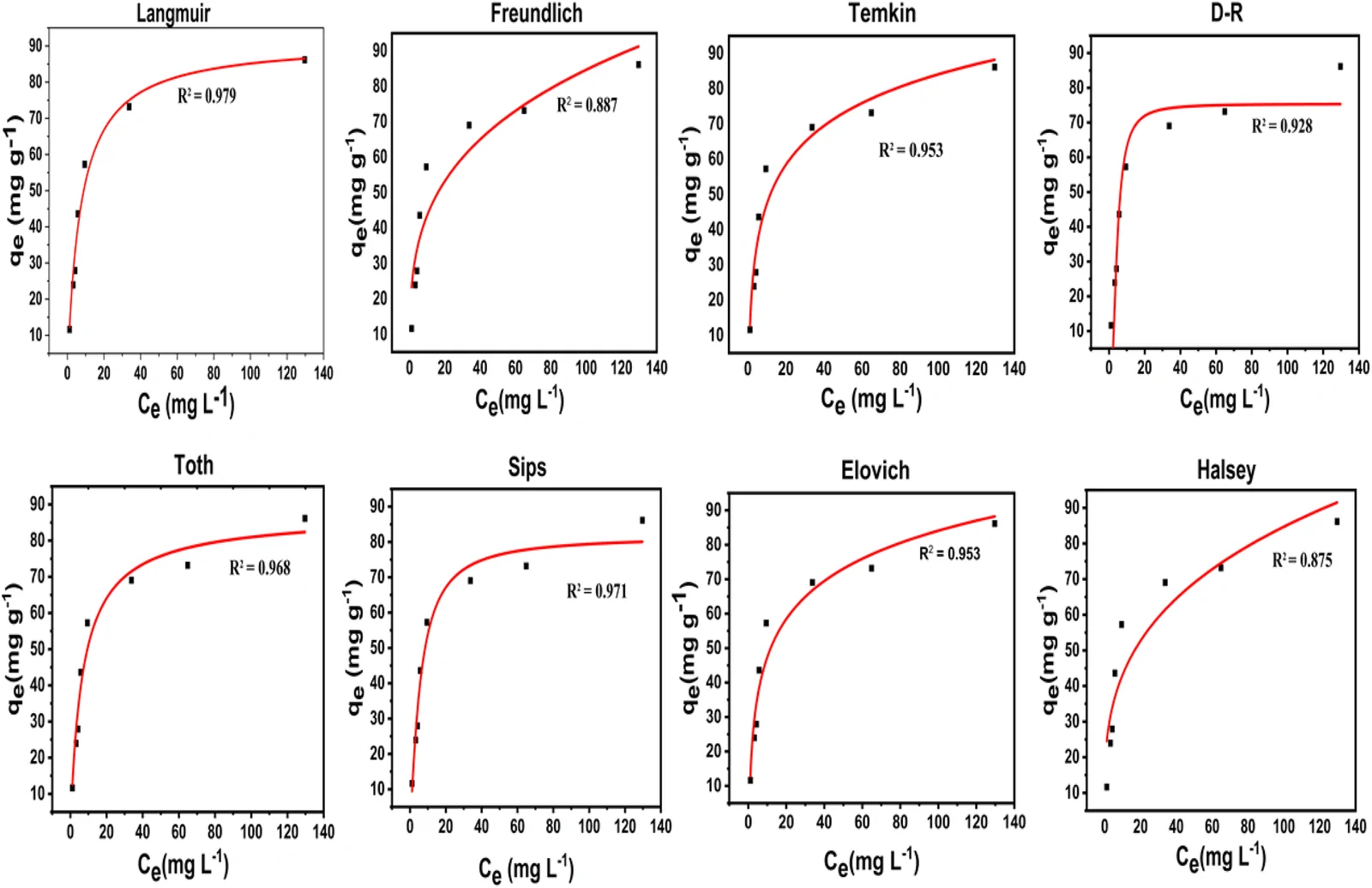
Isotherm plots showing the relationship between equilibrium adsorption capacity (*q*_e_) and equilibrium concentration (*C*_e_) obtained at 25 °C.

Eight isotherm models, Langmuir, Freundlich, Temkin, Dubinin–Radushkevich (D–R), Sips, Toth, Elovich, and Halsey, were applied to the equilibrium dataset. The corresponding qe–Ce relationships are presented in Fig. 17, while fitted parameters and statistical descriptors are compiled in Tables 4 and 5.

**Table 4:** Statistical parameters and goodness-of-fit ranking of isotherm models for tetracycline (TC) adsorption on La_9_–ACLP

Model	*R* ^2^	RMSE (mg g^−1^)	*χ* ^2^	AIC	ΔAIC	Ranking
Langmuir	0.9789	5.062	3.824	29.948	0.964	3
Freundlich	0.8871	8.343	15.78	37.94	8.956	6
Temkin	0.9534	5.357	5.535	30.85	1.870	4
Sips	0.9713	2.873	3.046	28.984	0	1
Toth	0.9684	4.414	3.461	29.757	0.773	2
Dubinin–Radushkevich	0.9287	6.628	4792.65	34.262	5.278	5
Elovich	0.9534	5.357	5.535	30.854	1.870	4
Halsey	0.8755	8.759	17.354	38.722	9.737	7

**Table 5:** Isotherm model parameters describing tetracycline (TC) adsorption on La_9_–ACLP

Model	Parameter	Value
Langmuir	*q*_max_ (mg g^−1^)	91.42
	*K*_L_ (L mg^−1^)	0.1354
Freundlich	*K*_f_ ((mg g^−1^)(L mg^−1^)^1/*n*^)	24.480
	*n*	3.472
Temkin	*A*_T_ (L g^−1^)	1.956
	*B*_T_ (J mol^−1^)	15.936
Sips	*q*_max_ (mg g^−1^)	81.756
	*K*_s_ (L mg^−1^)^*n*^	0.116
	*n*	1239
Toth	*q*_max_ (mg g^−1^)	82.921
	*K*_*t*_ (L mg^−1^)	0.156
	*t*	0.9892
Dubinin–Radushkevich	*q*_max_ (mg g^−1^)	75.394
	*B* (mol^2^ kJ^−2^)	19.872
Elovich	*K*_E_ (L mg^−1^)	31.17
	*B* (g mg^−1^)	0.0627
Hasley	*K*_H_ (mg g^−1^)	28 482.79
	*n*	3.349

• Statistical evaluation and model discrimination

Model performance was compared using a set of statistical indicators (*R*^2^, RMSE, *χ*^2^, and Akaike information criterion, AIC). No single metric fully captures model adequacy. The Langmuir equation reached a relatively high *R*^2^ value (0.9789), yet larger residual deviations persisted across parts of the concentration range, pointing to incomplete agreement between calculated and measured values.

The Sips equation provided the closest match to the dataset. Lower RMSE (2.873), reduced *χ*^2^ (3.046), and the minimum AIC (28.984; ΔAIC = 0) support this observation. The Toth model followed closely, with only a minor difference in AIC (ΔAIC = 0.773), placing both models within a comparable statistical range. The slight advantage of Sips likely reflects its adaptable form, which accommodates moderate deviations from ideal Langmuir behavior.

These comparisons also highlight a limitation of relying solely on *R*^2^, especially for heterogeneous systems where residuals are unevenly distributed. Joint interpretation of multiple statistical criteria provides a more robust basis for model selection.^55–57^

• Interpretation of adsorption characteristics

The strong agreement obtained with Sips and Toth points toward a surface that departs from ideal homogeneity. The equilibrium profile aligns more closely with a finite-capacity surface containing adsorption domains of different apparent energies.

The Sips heterogeneity parameter (*n* ≈ 1.239) reflects a moderate deviation from Langmuir assumptions. This parameter is best viewed as an empirical descriptor of surface non-uniformity rather than direct mechanistic evidence of multilayer formation. Such behavior is consistent with composite adsorbents where porous carbon frameworks coexist with dispersed La-based oxide phases, creating multiple adsorption environments.

The Toth model also reproduces the equilibrium data well, consistent with its suitability for mildly heterogeneous surfaces. The Freundlich equation remains useful at low surface coverage but lacks an explicit saturation term, which limits its applicability at higher concentrations. The Temkin model incorporates variations in adsorption energy, yet may oversimplify systems with broad or irregular energy distributions. Halsey and Elovich models yield weaker agreement under the present conditions, indicating a narrower range of applicability.^57^

These differences underscore the advantage of flexible models when the dataset spans a wide concentration interval.

Maximum capacities derived from Langmuir (91.42 mg g^−1^), Sips (81.76 mg g^−1^), and Toth (82.92 mg g^−1^) fall within a similar range. Although each model defines capacity differently, the convergence of these values supports internal consistency of the dataset rather than confirming any single model as universally valid.

The D–R model gives a mean adsorption energy below 8 kJ mol^−1^. Values in this range are commonly associated with relatively weak interactions. At the same time, the D–R model offers only an approximate energetic description and does not fully resolve the complexity of multifunctional composite surfaces.^58^ A cautious interpretation is therefore appropriate. Non-covalent interactions, such as electrostatic attraction, hydrogen bonding, and dispersion forces, likely play a major role, while localized stronger interactions, including coordination with La-containing sites, may also contribute.

### 3.5. Thermodynamic analysis of tetracycline adsorption onto La_9_–ACLP

**Fig. 18:**
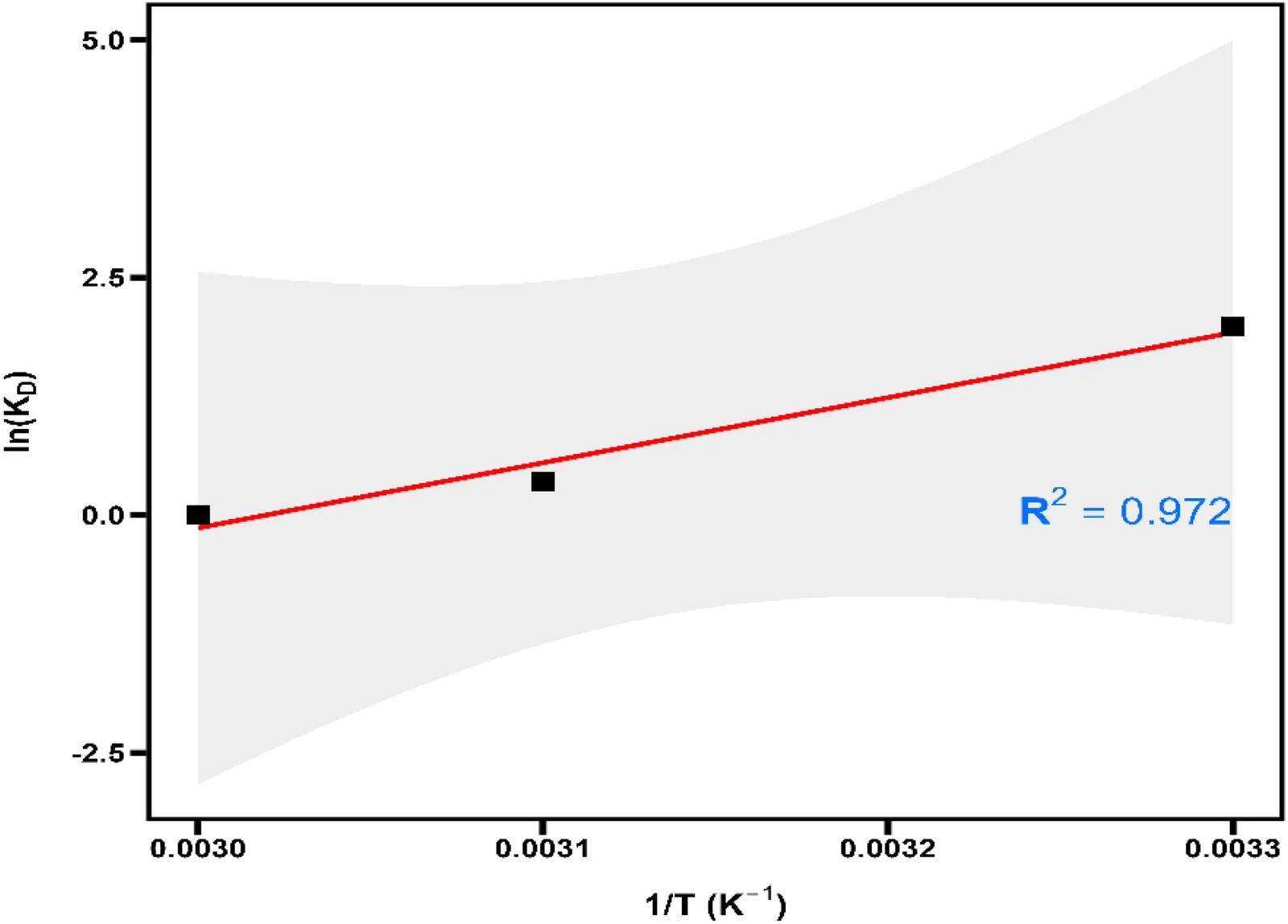
Van't Hoff plot for tetracycline (TC) adsorption onto La_9_–ACLP used for estimation of thermodynamic parameters (Δ*H*° and Δ*S*°). The shaded region represents the confidence interval of the linear regression fit.

Thermodynamic parameters for TC adsorption on La_9_–ACLP were calculated from equilibrium data obtained at different temperatures (Fig. 18 and Table 6). The Van't Hoff relationship between ln *K*_D_ and 1/*T* was used for estimation of Δ*G*°, Δ*H*°, and Δ*S*°. Because these parameters were derived from distribution coefficients, they should be regarded as operational thermodynamic values that depend partly on the selected equilibrium model and calculation approach.

**Table 6:** Thermodynamic parameters for tetracycline (TC) adsorption

*T* (K)	Δ*G*^0^ (kJ mol^−1^)	Δ*H*^0^ (kJ mol^−1^)	Δ*S*^0^ (kJ mol^−1^ K^−1^)
298	−5.7188	−56.945	−0.1719
313	−3.1403		
323	−1.4213		

Over the investigated temperature range (298–323 K), Δ*G*° values remained negative (−5.72 to −1.42 kJ mol^−1^), indicating that TC adsorption on La_9_–ACLP was thermodynamically favorable under the studied conditions. The decrease in adsorption favorability with increasing temperature is reflected by the less negative Δ*G*° values observed at higher temperature. The magnitude of Δ*H*° suggests that tetracycline adsorption on La_9_–ACLP likely involves multiple interaction pathways with different energetic contributions rather than being governed exclusively by weak dispersive interactions. At the same time, thermodynamic parameters alone are insufficient to unambiguously distinguish specific adsorption mechanisms and should therefore be interpreted together with the kinetic, equilibrium, and spectroscopic observations. The negative enthalpy change (Δ*H*° = −56.945 kJ mol^−1^) indicates that the adsorption process was exothermic, which agrees with the lower TC uptake observed at elevated temperature. Although the magnitude of Δ*H*° suggests contributions from more than one interaction pathway, enthalpy values alone cannot clearly differentiate between physisorption- and chemisorption-dominated behavior. The thermodynamic response is therefore more consistent with a heterogeneous adsorption environment involving several concurrent surface interactions rather than a single uniform adsorption pathway.^59,60^

Linear relationship between ln *K*_D_ and 1/*T* used to estimate thermodynamic parameters (Δ*H*° and Δ*S*°); shaded region indicates the confidence interval of the regression fit (*R*^2^ = 0.972).

The negative entropy change (Δ*S*° = −0.1719 kJ mol^−1^ K^−1^) suggests increased interfacial ordering during adsorption, consistent with the transfer of TC molecules from the aqueous phase to a more spatially constrained adsorbed state at the solid–solution interface.^61^ This entropy reduction may additionally reflect partial desolvation of TC molecules, restructuring of interfacial water, and enhanced organization within the adsorbed layer following surface association.

TC adsorption on La_9_–ACLP was spontaneous and exothermic over the studied temperature range. The negative entropy change suggests increased ordering at the solid–solution interface during adsorption.

The thermodynamic parameters also indicate that adsorption did not occur through a single uniform interaction pathway. Instead, the adsorption process likely involved several surface interactions with different energetic contributions. This interpretation agrees with the heterogeneous adsorption behavior observed from the kinetic and isotherm analyses.

### 3.6. Comparison with previously reported adsorbents

Table 7 places the adsorption performance of La_9_–ACLP in the context of representative lanthanum-containing and biomass-derived adsorbents reported for tetracycline removal. Comparison based solely on *q*_max_ may not fully reflect adsorbent performance because adsorption capacity depends not only on surface area but also on pore accessibility, surface chemistry, and the effectiveness of adsorption-active domains. Direct comparison should therefore be interpreted with caution because adsorption performance is strongly influenced by experimental conditions, including solution pH, ionic strength, initial tetracycline concentration, adsorbent dosage, contact time, temperature, and equilibrium modelling methodology. Accordingly, the comparison presented here is intended to provide performance context rather than establish superiority among different adsorbents.

**Table 7:** Comparison of tetracycline adsorption capacities, textural properties, adsorption mechanisms, and key structure–property characteristics of representative lanthanum-containing and biomass-derived adsorbents reported in the literature

Group	Adsorbent	BET SSA (m^2^ g^−1^)	*q*_max_ (mg g^−1^)	Main adsorption mechanism(s)	Advantages and limitations	Ref.
(A) Lanthanum-based systems	La-enriched TiO_2_–ZrO_2_	n.r.	52.6	Electrostatic attraction, surface complexation, photocatalytic degradation	Advantage: combines adsorption and photocatalytic degradation	62
					Limitation: pore structure optimization was not systematically addressed	
	La/Fe co-modified biochar (La/Fe@RSBC)	132.4	414.84	Hydrogen bonding, surface complexation, pore filling, electrostatic interactions, π–π electron donor–acceptor (EDA) interactions	Advantage: very high adsorption capacity; synergistic Fe/La sites provide abundant active centers; regenerable using H_2_O_2_	27
	La-modified bentonite/biochar composite	87.5	74.5	Ion exchange, electrostatic interactions	Advantage: high structural stability due to bentonite incorporation	23
					Limitation: relatively modest specific surface area	
	La_2_S_3_-modified S,N-doped biochar	214.3	248.8	π–π interactions, electrostatic attraction, photocatalytic activity	Advantage: high adsorption performance and multifunctionality	63
					Limitation: relatively complex synthesis procedure	
	La_9_–ACLP	264.82	91.4	Qualitative structure–property framework involving pore accessibility, hydrogen bonding, π-associated interactions, electrostatic effects, and possible interactions involving lanthanum-containing surface domains	Advantage: emphasizes optimization of pore accessibility during lanthanum incorporation rather than maximizing metal loading alone, providing mechanistic insight into the balance between active-site generation and pore accessibility.	This work
(B) Other adsorbent systems	Porous graphitic biochar	1645	1122.2	π–π interactions, pore filling, hydrogen bonding	Advantage: extremely high surface area and exceptional adsorption capacity.	47
					Limitation: requires high-temperature activation (800–1000 °C).	
	Maple leaf biochar	191.1	361.0	π–π interactions, hydrophobic interactions, pore filling	Advantage: high adsorption performance from naturally abundant leaf biomass.Limitation: strong dependence on pyrolysis temperature.	64
	Ball-milled biochar	58.7	197.3	Hydrogen bonding, π interactions, pore filling	Advantage: enhanced adsorbate accessibility through physical ball milling.	65
					Limitation: limited pore development.	
	Poultry manure biochar	47.91	65.0	Strong π–π and n–π interactions	Advantage: valorization of poultry waste; highly alkaline surface (pH ≈ 12.2).	66
					Limitation: moderate adsorption capacity.	
	Raw date palm waste	n.r.	29.16	π–π interactions, hydrogen bonding, van der Waals interactions, electrostatic attraction	Advantage: extremely low-cost adsorbent requiring no activation treatment.	67
					Limitation: low adsorption capacity.	
	Agricultural discarded material	n.r.	15.52	π–π EDA interactions, hydrogen bonding, partitioning	Advantage: produced at low pyrolysis temperature (300 °C), minimizing PAH emissions.	68
					Limitation: limited adsorption performance.	

This consideration is evident among lanthanum-based materials, whose reported adsorption capacities range from 52.6 to 414.84 mg g^−1^. La-enriched TiO_2_–ZrO_2_ achieved an adsorption capacity of 52.6 mg g^−1^ and was reported to remove tetracycline through electrostatic attraction, surface complexation, and photocatalytic processes.^62^ In contrast, La/Fe co-modified rice husk biochar (La/Fe@RSBC) reached 414.84 mg g^−1^ despite possessing a BET surface area of only 132.4 m^2^ g^−1^,^27^ whereas La_2_S_3_-modified S,N-doped biochar combined a BET surface area of 214.3 m^2^ g^−1^ with an adsorption capacity of 248.8 mg g^−1^.^63^ The La-modified bentonite/biochar composite exhibited a lower adsorption capacity of 74.5 mg g^−1^ despite its favorable structural stability.^23^ Collectively, these studies indicate that adsorption performance cannot be interpreted solely from BET surface area or lanthanum incorporation, but also depends on the accessibility and utilization of adsorption-active domains.

Against this background, La_9_–ACLP presents an interesting case. Although its adsorption capacity (91.4 mg g^−1^) is lower than that of several reported lanthanum-based adsorbents, it possesses the highest BET surface area within this group (264.82 m^2^ g^−1^). Such behavior suggests that adsorption is governed by more than the amount of accessible surface alone. The present results point to a balance between two competing effects: lanthanum incorporation generates additional adsorption-active domains, while excessive loading may progressively restrict access to internal pore networks through partial occupation of pore volume and transport pathways. The optimum performance observed for the La_9__ACLP (or La_9_) material appears to arise from this balance rather than from maximization of lanthanum content itself.

A similar pattern emerges among biomass-derived adsorbents. Porous graphitic biochar exhibited an exceptionally high adsorption capacity of 1122.2 mg g^−1^ together with a BET surface area of 1645 m^2^ g^−1^,^47^ whereas maple leaf biochar achieved 361.0 mg g^−1^ despite a much lower BET surface area of 191.1 m^2^ g^−1^.^64^. Ball-milled biochar provides another instructive example, reaching 197.3 mg g^−1^ with a BET surface area of only 58.7 m^2^ g^−1^.^65^ By comparison, poultry manure biochar, raw date palm waste, and agricultural discarded materials exhibited adsorption capacities ranging from 15.52 to 65.0 mg g^−1^.^66–68^ Together, these studies reinforce the view that adsorption capacity is not directly proportional to surface area and that adsorption-site accessibility and utilization are equally important.

A simplified comparison of adsorption density (*q*_max_/BET) further illustrates this point, although the parameter should be interpreted cautiously because BET surface area does not necessarily represent adsorption-accessible surface area. It is introduced only as a simplified descriptor of surface utilization efficiency and should not be interpreted as a rigorous mechanistic metric or used for quantitative ranking among different adsorbents. La_9_–ACLP exhibits an adsorption density of approximately 0.35 mg m^−2^, whereas La/Fe@RSBC and ball-milled biochar reach approximately 3.13 and 3.36 mg m^−2^, respectively. These differences suggest that the effectiveness with which adsorption sites are utilized may be as important as their absolute abundance.

The textural properties of La_9_–ACLP provide additional context. Nitrogen adsorption–desorption analysis revealed a predominantly mesoporous structure with a BET surface area of 264.82 m^2^ g^−1^, a pore volume of 0.1175 cm^3^ g^−1^, and an average pore diameter of 6.37 nm. Such mesopores facilitate molecular transport toward internal adsorption regions while maintaining access to adsorption-active domains. Preserving these transport pathways after lanthanum incorporation therefore appears to be important because additional adsorption-active domains are expected to contribute only when they remain accessible to adsorbate molecules.

This interpretation is consistent with the broader adsorption behaviour observed in the present study. The kinetic analysis suggests that adsorption cannot be adequately described by a single elementary process, whereas the superior fitting of the Sips and Toth models indicates adsorption on energetically heterogeneous surfaces. Considered together with the mesoporous structure, the increase in pH_p_zc from 5.5 to 7.6 following lanthanum incorporation, and the spectroscopic evidence discussed in Section 3.7, these observations support a qualitative interpretation in which pore accessibility, surface chemistry, and lanthanum-containing surface domains collectively influence tetracycline uptake.

The studies summarized in Table 7 converge on a common conclusion: neither BET surface area, lanthanum loading, nor adsorption capacity alone provides an adequate description of adsorption behaviour. Instead, tetracycline uptake reflects the combined influence of pore architecture, molecular transport, surface functionality, and adsorption-site chemistry. Within this context, the principal scientific contribution of the present work lies in establishing an experimentally supported qualitative structure–property framework indicating how controlled lanthanum incorporation influences both adsorption-active domains and pore accessibility within the scope of the available experimental evidence.

### 3.7. Proposed adsorption mechanism of tetracycline onto La_9_–ACLP

#### 3.7.1 Mechanistic overview

Adsorption of tetracycline (TC) by La_9_–ACLP is best interpreted as a heterogeneous interfacial process arising from multiple cooperative interactions rather than a single dominant adsorption mechanism. The following discussion integrates complementary evidence from structural characterization, surface analysis, adsorption modelling, and thermodynamic evaluation while recognizing that no individual characterization technique can independently verify molecular-scale adsorption interactions. Accordingly, the proposed adsorption mechanism should be regarded as a qualitative interpretation derived from the collective experimental observations rather than direct molecular-scale evidence.

Throughout this discussion, the term adsorption interface refers to the collective physicochemical characteristics of the adsorbent surface, including pore accessibility, surface chemistry, structural heterogeneity, and interfacial charge behaviour, as inferred from the complementary characterization results. It does not imply direct observation of molecular adsorption events or specific adsorbate–surface interactions.

The characterization results presented in Section 3.1 collectively characterize the physicochemical context of the adsorption interface. XRD, SEM, TEM, and EDS collectively indicate that finely dispersed La_2_O_3_ nanoparticles are distributed throughout the preserved turbostratic carbon framework. FT-IR indicates the retention of oxygen-containing functional groups, whereas XPS identifies the presence of surface-exposed La^3+^ species. BET analysis and pH_p_zc measurements further show that lanthanum modification is associated with changes in pore accessibility and interfacial charge characteristics without disrupting the hierarchical porous architecture. Collectively, these observations describe a chemically heterogeneous adsorbent surface and provide the structural basis for interpreting the adsorption process.

Each characterization technique provides complementary information regarding a different physicochemical aspect of the adsorption interface. Considered together with the adsorption isotherms, kinetic analyses, and thermodynamic results, these datasets provide a coherent basis for discussing possible adsorption interactions while avoiding interpretations beyond the available experimental evidence.

**Fig. 19:**
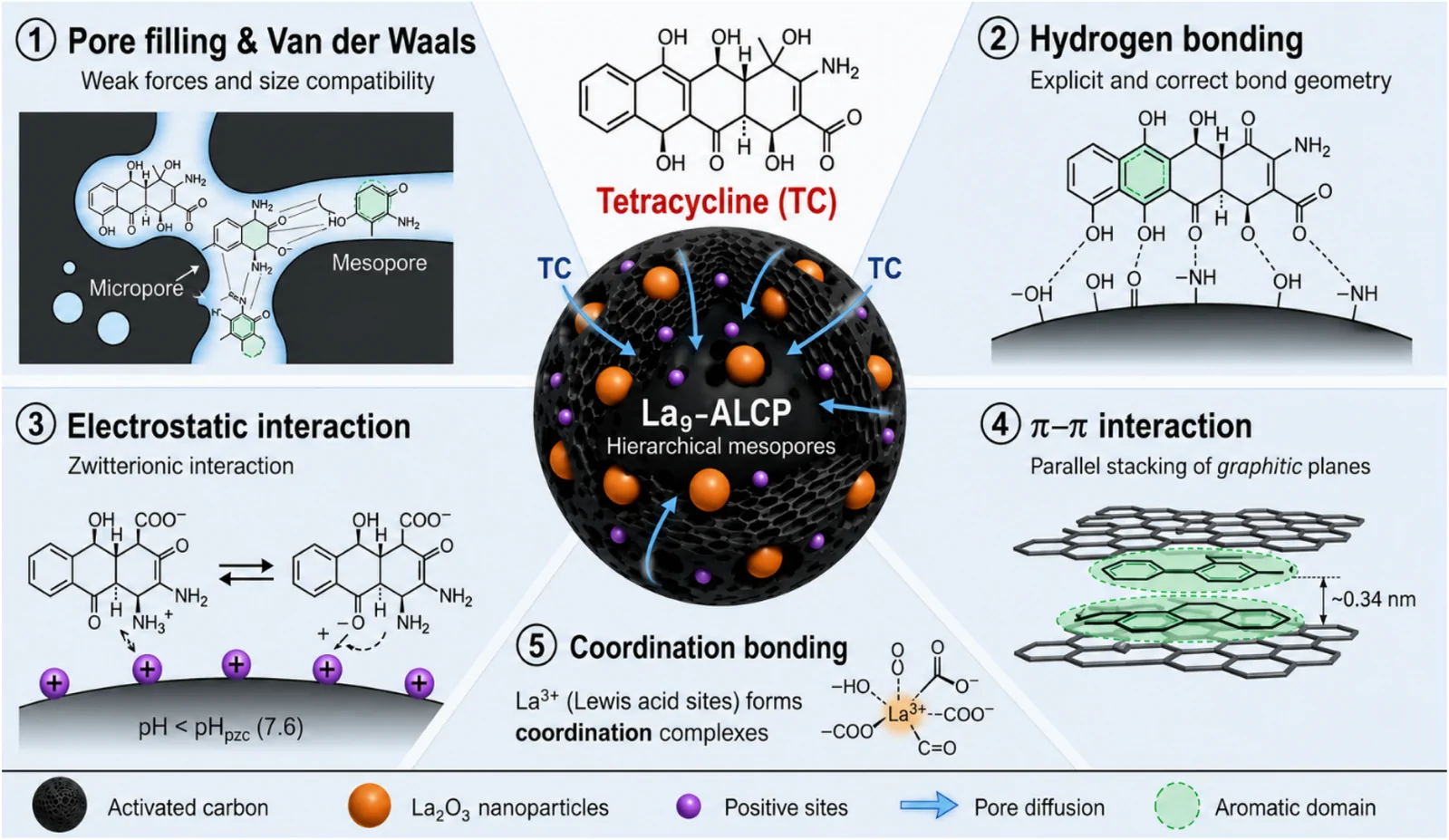
Schematic illustration of the multi-pathway adsorption mechanism of tetracycline (TC) onto La_9_–ACLP.

Within this framework, and in conjunction with the complementary structural characterization, adsorption modelling, and thermodynamic evidence, TC adsorption is interpreted as the combined outcome of pore accessibility, hydrogen bonding, π-associated interactions, pH-dependent electrostatic effects, lanthanum-containing surface interactions, and non-specific dispersion forces. The relative contribution of each interaction is expected to vary with solution chemistry, adsorbate concentration, temperature, and surface coverage. The following sections examine the experimental observations that are consistent with the plausibility of each proposed interaction before integrating them into the qualitative adsorption framework summarized in Fig. 19.

#### 3.7.2 Structural foundation established by characterization

Before discussing the individual adsorption interactions, it is first necessary to consider how lanthanum modification changes the physicochemical characteristics of the adsorbent. Rather than representing isolated observations, the complementary characterization results collectively describe structural evolution at the crystallographic, morphological, surface-chemical, and pore-structural levels, thereby characterizing the physicochemical context encountered by dissolved tetracycline (TC) molecules.

XRD indicates that lanthanum modification introduces finely dispersed crystalline La_2_O_3_ while preserving the turbostratic carbon framework. This interpretation is supported by SEM, TEM, and EDS, which show that La_2_O_3_ nanoparticles are homogeneously distributed throughout the porous carbon matrix rather than concentrated in isolated regions. Collectively, these observations indicate a homogeneous distribution of La_2_O_3_ nanoparticles throughout the porous carbon framework while preserving the underlying carbon scaffold.

FT-IR indicates the retention of oxygen-containing functional groups following modification, whereas XPS identifies the presence of surface-exposed La^3+^ species associated with La_2_O_3_. Together, these observations suggest that lanthanum modification is associated with changes in the local surface chemistry while largely preserving the original carbon framework. Accordingly, the adsorbent surface is characterized by aromatic carbon domains, oxygen-containing functional groups, and surface-exposed lanthanum species, collectively providing a chemically heterogeneous adsorption environment. Although this structural framework characterizes the physicochemical properties of the adsorbent, it does not by itself establish the molecular interactions responsible for TC adsorption. Rather, it defines the structural and surface characteristics that constrain the range of adsorption interactions considered in the following discussion.

These chemical modifications occur concurrently with changes in the pore structure. BET analysis shows that the hierarchical porous architecture is retained after lanthanum incorporation despite moderate decreases in surface area and pore volume, which is consistent with localized coverage of accessible pore surfaces by La_2_O_3_ nanoparticles rather than collapse of the carbon framework. In parallel, the increase in pH_p_zc indicates that lanthanum modification is associated with changes in the interfacial acid–base characteristics of the adsorbent. Viewed collectively, these physicochemical observations indicate an adsorption environment that is expected to influence TC adsorption while maintaining access to the interconnected pore network.

Overall, the characterization results suggest that the observed changes in adsorption behaviour are associated with the concurrent modification of pore accessibility, surface chemistry, and interfacial charge behaviour while preserving the carbon scaffold. These collectively modified physicochemical characteristics provide the context within which the possible adsorption interactions discussed in the following sections should be interpreted. Accordingly, the possible adsorption interactions are interpreted as complementary contributors to the overall adsorption behaviour rather than as mutually exclusive adsorption mechanisms.

Although lanthanum incorporation systematically modifies both pore structure and surface chemistry, the present experimental dataset does not permit their respective contributions to adsorption behaviour to be quantified independently. Accordingly, the following discussion considers the possible adsorption interactions by integrating complementary structural characterization, surface analyses, adsorption modelling, and thermodynamic evidence. None of the individual adsorption interactions discussed below should therefore be regarded as being independently verified by a single characterization technique. Instead, the proposed qualitative structure–property framework is interpreted from the collective experimental observations rather than representing a quantitative decomposition of the contributions of individual interactions.

#### 3.7.3 Pore accessibility and mass transport

Successful adsorption requires molecular transport from the bulk solution to the internal pore network of the adsorbent. Accordingly, pore accessibility influences whether dissolved TC molecules can access internal surface regions where subsequent adsorption interactions may occur. Mass transport should therefore be regarded as a structural prerequisite for adsorption rather than an adsorption mechanism itself.

BET analysis indicates that lanthanum modification alters the pore structure without disrupting the hierarchical porous architecture generated during H_3_PO_4_ activation. Although the BET surface area and pore volume decrease after modification, the preserved mesoporous network suggests that interconnected transport pathways remain available. These changes are more consistent with localized coverage of accessible pore surfaces by La_2_O_3_ nanoparticles than with structural collapse. However, these structural changes alone do not adequately explain the observed adsorption behaviour, indicating that complementary changes in surface chemistry and interfacial charge should also be considered.

The adsorption kinetics are consistent with this structural interpretation. Rapid initial uptake is consistent with efficient external mass transfer, whereas the subsequent decrease in adsorption rate reflects progressive diffusion toward less accessible internal regions. Similarly, the Weber–Morris intraparticle diffusion model indicates that intraparticle diffusion contributes to, but does not solely determine, the overall adsorption rate.

Pore accessibility alone, however, cannot explain the overall adsorption behaviour. The relatively low adsorption energy obtained from the Dubinin–Radushkevich model (<8 kJ mol^−1^) is consistent with contributions from weak non-specific interactions, including pore filling and dispersion forces.^69^ Likewise, the preference for heterogeneous equilibrium models such as the Sips and Toth isotherms suggests adsorption over surface regions with different energetic characteristics rather than a single class of adsorption sites. Accordingly, pore accessibility provides the structural conditions for adsorption but should not itself be interpreted as the dominant adsorption mechanism. The possible adsorption interactions associated with these physicochemical characteristics are discussed in the following sections.

#### 3.7.4 Hydrogen-bonding interactions

Hydrogen bonding is considered a plausible contributor to TC adsorption once the molecules gain access to the adsorbent surface described above. The FT-IR spectra (Fig. 6 and Table 1) show attenuation or disappearance of the free –OH and –OH/N–H stretching bands after adsorption together with a slight shift of the broad band near 3120 cm^−1^, indicating changes in the local hydrogen-bonding environment following TC uptake. These spectral variations are consistent with changes in the local chemical environment that may arise from interactions involving the hydroxyl, amide, and carbonyl functionalities of TC and oxygen-containing surface groups on the adsorbent.^70,71^

This interpretation is further supported by the preservation of oxygen-containing surface species after lanthanum modification. FT-IR indicates that oxygen-containing functional groups remain after modification, whereas XPS identifies surface oxygen species associated with both the carbon matrix and lanthanum oxide, suggesting that potential hydrogen-bond donor and acceptor sites are present before adsorption. These observations support the plausibility of hydrogen-bonding interactions but do not constitute direct evidence of hydrogen-bond formation during adsorption. Accordingly, hydrogen bonding is interpreted as one of several possible interactions contributing to TC adsorption rather than as an independently verified dominant mechanism.

#### 3.7.5 π-Associated interactions

In addition to hydrogen bonding, the preserved aromatic framework of the activated carbon provides a structural basis for possible π-associated interactions with TC. XRD indicates that lanthanum incorporation preserves the turbostratic carbon framework, while the minor changes observed in the C 1 s XPS spectrum suggest that the aromatic carbon domains remain largely unchanged after modification. Collectively, these observations suggest that aromatic carbon domains capable of supporting π-associated interactions are expected to remain available on the adsorbent surface before adsorption. The extended conjugated ring system of TC makes such interactions chemically plausible from a structural perspective when these aromatic domains are accessible, consistent with previous studies reporting π-associated interactions between TC and condensed aromatic domains in biochar- and activated-carbon-based adsorbents.^36,72^

The available characterization data, however, do not allow π–π stacking to be distinguished unambiguously from other non-covalent interactions that may occur simultaneously during adsorption. Accordingly, π-associated interactions are interpreted as one of several possible contributors to the overall adsorption behaviour. Their potential contribution should therefore be considered together with hydrogen bonding, electrostatic interactions, and lanthanum-associated surface interactions within the qualitative structure–property framework proposed in this study.

#### 3.7.6 Electrostatic interactions

Electrostatic interactions are expected to influence TC adsorption by regulating the approach of dissolved molecules to the adsorbent surface rather than acting as the sole driving force for adsorption. This contribution is inherently dependent on solution pH because both the surface charge of the adsorbent and the speciation of TC vary with the surrounding chemical environment.

The increase in pH_pzc_ from 5.5 for ACLP to 7.6 following lanthanum incorporation indicates a substantial shift in the surface-charge characteristics, thereby modifying the electrostatic conditions governing adsorbent–solution interactions. Depending on solution pH, electrostatic attraction or repulsion may either facilitate or hinder molecular access to the adsorbent surface while acting in concert with hydrogen bonding and π-associated interactions.^72^ This behaviour suggests that electrostatic effects influence the likelihood of adsorbate–surface contact rather than directly determining the adsorption mechanism itself.

The present results do not permit the electrostatic contribution to be isolated quantitatively from the other intermolecular interactions discussed above. Instead, the pH_p_zc measurements indicate that lanthanum modification alters the interfacial charge characteristics, thereby changing the electrostatic conditions under which adsorption occurs. Accordingly, electrostatic interactions are interpreted as a pH-dependent contributor within the qualitative structure–property framework proposed in this study rather than as an independently dominant adsorption mechanism.

#### 3.7.7 Lanthanum-associated surface interactions

Unlike pristine ACLP, lanthanum-modified carbon exhibits chemically distinct surface characteristics associated with dispersed La_2_O_3_ nanoparticles. Pre-adsorption XPS analysis identifies lanthanum predominantly as oxidized La^3+^ species associated with La_2_O_3_, while FT-IR confirms the presence of La–O bonds following modification. Together with the homogeneous distribution of La_2_O_3_ nanoparticles observed by SEM, TEM, and EDS, these observations indicate the presence of surface-associated La_2_O_3_ species that are potentially available for interaction with dissolved TC molecules.

The oxygen-containing functional groups present in TC provide a structural basis for localized interactions with surface-associated lanthanum species. Such interactions are chemically plausible in light of previous reports that lanthanide oxides interact with oxygen-donor functionalities through Lewis acid–base interactions under appropriate solution conditions and therefore may interact with TC through similar interaction pathways.^27,73,74^ However, the present characterization data do not directly demonstrate the formation of inner-sphere surface complexes or specific La–TC coordination during adsorption because the XPS analysis represents the initial surface chemical state before adsorption. Instead, the available evidence suggests that lanthanum modification is associated with changes in the local surface chemistry, producing chemically distinct surface characteristics that may contribute cooperatively with hydrogen bonding, π-associated interactions, and electrostatic effects. Accordingly, the proposed role of lanthanum should be interpreted as an inference derived from complementary characterization and adsorption evidence rather than direct observation of La–TC coordination.

Accordingly, the enhanced adsorption performance of La_2_O_3_–ACLP should not be attributed solely to the introduction of lanthanum-associated surface characteristics. Rather, lanthanum modification alters the physicochemical characteristics of the adsorbent surface, thereby altering the adsorption environment within the qualitative structure–property framework proposed in this study.

#### 3.7.8 Integrated adsorption framework

The preceding analyses collectively indicate that the adsorption behaviour of tetracycline (TC) cannot be adequately interpreted in terms of any single adsorption interaction. Rather, the complementary structural characterization, adsorption modelling, and thermodynamic analyses are consistent with an integrated adsorption framework in which molecular transport, surface heterogeneity, and interfacial physicochemical properties collectively contribute to the overall adsorption behaviour. Accordingly, the framework summarized in Fig. 19 should be interpreted as a qualitative synthesis of the complementary experimental evidence rather than as direct verification of individual adsorption interactions.

Following lanthanum modification, the available characterization data indicate concurrent changes in pore structure, surface chemistry, and interfacial charge characteristics while preserving the hierarchical porous carbon framework. Together, these observations are consistent with a chemically heterogeneous adsorption environment in which dissolved TC molecules are likely to encounter different local physicochemical conditions during diffusion through the pore network. Consequently, the observed adsorption behaviour is more appropriately interpreted as arising from the combined influence of structural accessibility and interfacial physicochemical properties than from any isolated adsorption interaction.

The adsorption models are likewise consistent with this integrated interpretation. Kinetic analyses indicate sequential mass transfer followed by surface-associated processes, reflecting the progressive accessibility of heterogeneous adsorption regions. Similarly, the preference for the heterogeneous Sips and Toth isotherms indicates adsorption over surface regions with different energetic characteristics rather than a single energetically uniform surface. Furthermore, the mean adsorption energy estimated from the Dubinin–Radushkevich model (<8 kJ mol^−1^) is consistent with weak non-specific interactions operating alongside the intermolecular interactions discussed in Sections 3.7.3–3.7.7.

The thermodynamic analyses further complement this qualitative interpretation. The spontaneous adsorption process, together with the corresponding enthalpic and entropic changes, is consistent with adsorption involving multiple physicochemical processes within a chemically heterogeneous adsorption environment rather than a single elementary interaction. Accordingly, the observed thermodynamic behaviour should be interpreted as an emergent property of the modified adsorbent, in which multiple intermolecular interactions may jointly contribute to the overall adsorption energetics without implying that any individual interaction has been independently verified.

Overall, the collective experimental observations suggest that the improved adsorption performance of La_9_–ACLP is associated with concurrent structural and surface-chemical modifications following lanthanum incorporation. These complementary changes establish the physicochemical context within which multiple adsorption interactions may operate cooperatively. XPS analysis characterized the initial chemical state of lanthanum prior to adsorption, confirming the presence of surface-associated La–O species, but does not reveal how lanthanum species evolve during adsorption. Instead, the adsorption behaviour is qualitatively interpreted from complementary structural characterization, pH_pzc_ measurements, pH-dependent adsorption behaviour, and kinetic and equilibrium analyses. Within the scope of the available experimental evidence, hydrogen bonding, π-associated interactions, electrostatic effects, and possible interactions involving surface-associated lanthanum species are considered plausible contributors to TC adsorption. Because post-adsorption surface-sensitive characterization was not performed, neither La–TC coordination nor the evolution of lanthanum species during adsorption can be directly verified. Accordingly, Fig. 19 should be regarded as a conceptual representation of the qualitative structure–property framework proposed in this study, integrating the available experimental evidence rather than depicting a directly verified molecular adsorption mechanism.

Accordingly, the proposed adsorption framework should be regarded as a qualitative interpretation supported by complementary physicochemical evidence rather than direct verification of adsorption affinity, active-site distribution, or interfacial chemical environments.

### 3.8. Structural evolution and reusability after repeated adsorption–desorption cycles

#### 3.8.1 Structural evolution after four cycles

**Fig. 20:**
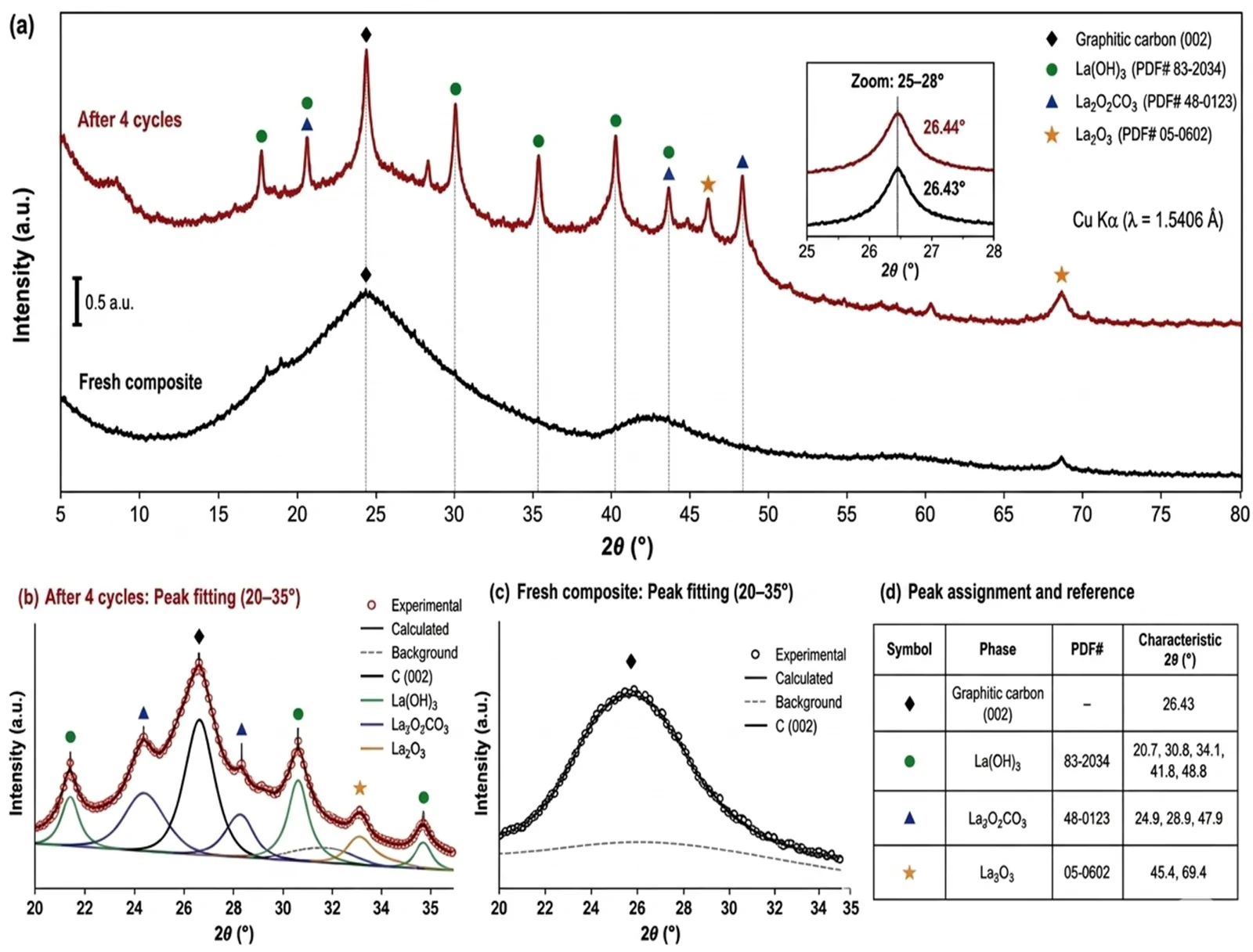
(a) XRD patterns of fresh La_2_O_3_–ACLP and the sample recovered after four adsorption–desorption cycles. (b) Peak deconvolution in the 20–35° region. (c) Enlarged view of the carbon (002) reflection showing negligible peak shift. (d) Comparison with reference diffraction patterns of La_2_O_3_, La(OH)_3_ and La_2_O_2_CO_3_.

Structural stability after repeated operation was assessed by comparing the XRD patterns of the fresh composite and the material recovered after four adsorption–desorption cycles (Fig. 20). The fresh sample was dominated by a broad reflection centered at 2*θ* = 26.43°, characteristic of the (002) plane of poorly ordered graphitic carbon. Peak deconvolution in the 20–35° region further revealed a predominantly low-crystallinity carbon framework without clearly resolved reflections attributable to crystalline lanthanum-containing phases within the detection limit of XRD. After four cycles, the broad carbon-related reflection remained essentially unchanged, shifting only from 26.43° to 26.44° (Fig. 20c). The absence of appreciable peak displacement or major distortion of the diffraction profile suggests that the carbon framework retained its crystallographic characteristics during repeated adsorption–regeneration treatment. For completeness, the unprocessed XRD profiles are provided in Fig. S3 (SI) and exhibit a comparable overall diffraction pattern before and after cycling.

Several additional reflections nevertheless became discernible in the recycled sample. Diffraction features at approximately 20.7°, 30.8°, 34.1°, 41.8°, and 45.6° are consistent with La(OH)_3_ (PDF# 83-2034), whereas peaks at 24.9°, 28.4°, and 47.9° are consistent with diffraction features of La_2_O_2_CO_3_ (PDF# 48-0123). Weak reflections near 45.4° and 69.4° may be associated with residual La_2_O_3_ (PDF# 05-0602). These assignments are supported by the peak-fitting analysis and reference diffraction patterns presented in Fig. 20.

The emergence of hydroxylated and oxycarbonate lanthanum phases indicates localized surface transformation of La_2_O_3_ during repeated aqueous operation. Such phase evolution is consistent with the progressive hydration of exposed lanthanum oxide domains during repeated aqueous operation, followed by partial carbonation through interaction with dissolved or atmospheric CO_2_. Similar phase evolution has been reported for lanthanum-based adsorbents under aqueous conditions.^75^

Overall, the diffraction data support preservation of the carbon framework after four adsorption–desorption cycles while revealing limited surface phase evolution involving the lanthanum-containing surface domains. The coexistence of framework stability and localized surface transformation highlights the dynamic evolution of the lanthanum-containing surface domains during repeated operation. The observed changes are consistent with environmentally induced transformation of surface lanthanum species rather than degradation of the underlying carbon scaffold. It is important to recognize that post-regeneration XRD and XPS analyses provide information only on the solid-state evolution of lanthanum-containing phases and do not quantify dissolved lanthanum release. Therefore, the extent of lanthanum release under adsorption and regeneration conditions remains to be established through future ICP-OES/MS measurements before the environmental stability of lanthanum under these conditions can be fully assessed.

#### 3.8.2 Reusability after repeated four cycles

**Fig. 21:**
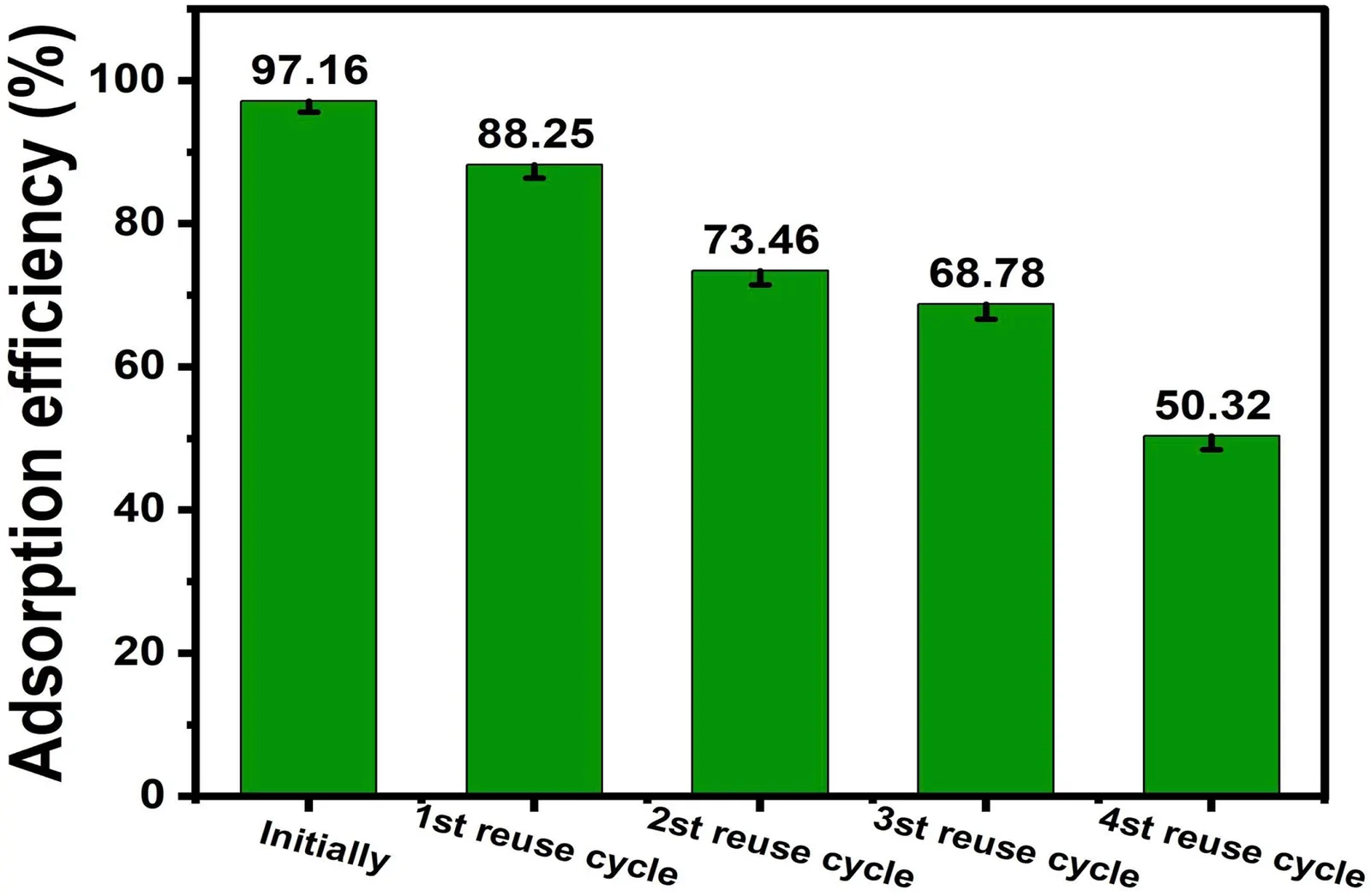
Adsorption efficiency of La_9_–ACLP during four consecutive adsorption–desorption cycles. Error bars represent the standard deviation (*n* = 3).

The reusability of La_9_–ACLP was evaluated over four consecutive adsorption–desorption cycles (Fig. 21).

The adsorption efficiency decreased progressively from 97.16% for the fresh adsorbent to 88.25%, 73.46%, 68.78%, and 50.32% after the first, second, third, and fourth cycles, respectively. The XRD results discussed above provide structural context for interpreting the observed cycle-by-cycle adsorption performance rather than direct evidence of reusability. Although the turbostratic carbon framework remained structurally preserved after repeated operation, localized phase evolution involving the lanthanum-containing surface domains was observed. The gradual loss in adsorption efficiency is therefore more reasonably attributed to the combined influence of incomplete desorption of strongly retained TC and the progressive evolution of the lanthanum-containing adsorption interface. In contrast, the XRD results provide little evidence that deterioration of the carbon scaffold is responsible for the observed decline. Despite the progressive decline in adsorption efficiency, approximately 50% of the initial tetracycline removal efficiency was retained after four regeneration cycles, indicating that the adsorbent maintained appreciable adsorption performance under the regeneration conditions employed.

### 3.9. Environmental implications

The present material integrates agricultural biomass valorization with water-treatment applications by converting locally available biomass residues into a functional carbon adsorbent. This approach promotes productive utilization of agricultural waste while providing a potentially sustainable alternative to fully synthetic adsorbents. The relatively simple preparation route and the abundance of biomass feedstocks may facilitate future development of decentralized or modular wastewater treatment technologies.

Tetracycline remains an environmental concern because of its persistence and its contribution to antimicrobial resistance (AMR). Within this context, La_9_–ACLP should be regarded as a complementary treatment material rather than a standalone remediation technology. The concentration range investigated here is more representative of concentrated antibiotic-containing waste streams, such as pharmaceutical and livestock effluents, than highly diluted natural waters.

From an operational perspective, XRD characterization after repeated adsorption–desorption cycles demonstrated preservation of the turbostratic carbon framework while revealing localized phase evolution of the lanthanum-containing active domains, consistent with progressive hydration and partial carbonation under aqueous conditions. Although adsorption performance gradually declined during regeneration, the preserved carbon framework suggests that this decrease was more likely associated with progressive evolution of the lanthanum-containing adsorption interface and incomplete desorption of strongly retained tetracycline than with deterioration of the carbon scaffold. These findings indicate that the biomass-derived carbon framework remains structurally resilient during repeated operation.

Despite these encouraging results, broader sustainability considerations, including lanthanum sourcing, resource recovery, and the potential release of lanthanum species during prolonged operation, should be incorporated into future assessments. Future studies should also address the regeneration and management of spent adsorbents to minimize secondary environmental impacts and maximize resource recovery. Integrating adsorption performance with regeneration efficiency, long-term structural stability, spent adsorbent management, and life-cycle sustainability will be essential for evaluating the practical feasibility of lanthanum-modified biomass-derived adsorbents in large-scale water-treatment applications.

### 3.10. Limitations and future perspectives

Several limitations should be considered before extending the present findings to practical wastewater treatment. First, the adsorption experiments were conducted in simplified aqueous solutions, whereas real wastewaters contain competing ions, dissolved organic matter, suspended solids, and fluctuating pH conditions that may influence adsorption behavior. Validation using real wastewater and continuous-flow systems is therefore required.

The long-term stability of lanthanum within the composite remains uncertain because lanthanum leaching was not quantified. Future studies should include ICP-OES or ICP-MS analyses to evaluate lanthanum release under both adsorption and regeneration conditions, allowing direct assessment of environmental safety and material stability.

Although XPS characterization of the fresh lanthanum-modified composite established the initial chemical states of the lanthanum-containing surface domains, post-adsorption and post-regeneration XPS analyses were not performed. Consequently, the adsorption-induced evolution of surface lanthanum species and their interactions with tetracycline could not be directly resolved. While XRD after repeated adsorption–desorption cycles demonstrated that the principal diffraction features associated with the carbon matrix and lanthanum-containing crystalline phases remained largely unchanged, complementary techniques such as post-adsorption XPS, EXAFS, or *in situ* spectroscopy are required to directly elucidate adsorption configurations and interfacial transformations.

Future investigations should integrate cycle-by-cycle adsorption measurements with post-regeneration structural characterization to establish clearer correlations between structural evolution and adsorption performance. Optimization of regeneration strategies and validation under environmentally relevant conditions will also be important for practical implementation. Therefore, conclusions regarding environmental applicability should be interpreted within the scope of these limitations. Despite these limitations, the present study establishes a mechanistic framework linking lanthanum incorporation, pore accessibility, surface chemistry, and adsorption behavior, providing a foundation for future optimization and validation of lanthanum-modified adsorbents for practical water-treatment applications.

## 4. Conclusion

Incorporation of La_2_O_3_ into hydrothermally derived activated carbon simultaneously modified pore accessibility and interfacial adsorption chemistry while preserving the structural integrity of the biomass-derived carbon framework. Among the investigated materials, La_9_–ACLP achieved the optimal balance between the generation of lanthanum-containing adsorption domains and the preservation of mesoporous transport pathways, resulting in the highest tetracycline adsorption performance.

Tetracycline adsorption arose from the synergistic contributions of pore filling, hydrogen bonding, π–π electron donor–acceptor interactions, electrostatic interactions, and localized lanthanum-associated surface interactions. Lanthanum incorporation further modified the surface acid–base characteristics, pH_pzc_, and La–O-containing surface environments, thereby regulating adsorption under circumneutral conditions. Collectively, the kinetic, equilibrium, thermodynamic, and complementary physicochemical characterization results support a qualitative interpretation in which adsorption appears to be governed by the interplay between structural accessibility and adsorption affinity rather than by a single dominant mechanism.

Repeated adsorption–desorption cycles preserved the turbostratic carbon framework while inducing only localized evolution of the lanthanum-containing surface domains, consistent with progressive hydration and partial carbonation under aqueous conditions. The gradual decline in adsorption performance is therefore more plausibly associated with evolution of the lanthanum-containing adsorption interface than with deterioration of the underlying carbon scaffold.

More fundamentally, this study demonstrates that the improved adsorption performance of lanthanum-functionalized biomass-derived carbon is associated with systematic modification of the interfacial adsorption environment rather than solely with an increase in the abundance of adsorption-active sites. The results suggest that lanthanum incorporation modifies structural accessibility, surface chemistry, and interfacial charge characteristics, thereby establishing chemically heterogeneous adsorption environments that cooperatively regulate tetracycline adsorption. These findings provide a basis for the rational design of lanthanum-functionalized carbon adsorbents, highlighting that balancing structural accessibility with adsorption affinity may be as important as increasing the abundance of adsorption-active sites. Because the present adsorption experiments were performed using model aqueous solutions, the influence of competing inorganic ions and dissolved natural organic matter on adsorption performance remains to be established. Future studies should quantitatively evaluate lanthanum leaching under adsorption and regeneration conditions using ICP-OES/MS, together with post-regeneration surface evolution, continuous-flow operation, and adsorption performance in environmentally relevant water matrices. Quantitative assessment of lanthanum release will be essential for confirming the long-term environmental compatibility and practical applicability of the material. Such investigations will further support the rational design of biomass-derived adsorbents in which interfacial chemistry and pore architecture are jointly optimized for the sustainable removal of emerging contaminants.

## Author contributions

Tra Huong Do conceived the study, designed the methodology, and drafted the manuscript. Truong Xuan Vuong contributed to data interpretation and led the writing and revision of the manuscript. Thi To Loan Nguyen., Van Nhuong Vu, Xuan Truong Mai, and Thi Hue Tran contributed equally to data acquisition, analysis, and critical revision of the manuscript. All authors discussed the results and approved the final version of the manuscript.

## Conflicts of interest

The authors declare no conflicts of interest.

## Data Availability

The data supporting this article are provided in the supplementary information (SI). Additional information is available from the corresponding author upon reasonable request. Supplementary information: comparative screening of lanthanum-modified activated carbons for tetracycline removal (Fig. S1), HPLC chromatograms and calibration data for tetracycline quantification (Fig. S2), and the raw XRD patterns of fresh and regenerated La_2_O_3_–ACLP supporting the diffraction analysis presented in the main text (Fig. S3). See DOI: https://doi.org/10.1039/d6ra05083b.
